# How a Single Atom Influences the Spatiotemporal Response of Flexible MOFs: Insights from Theory and Experiment

**DOI:** 10.1021/jacs.5c02552

**Published:** 2025-06-16

**Authors:** Szymon K Sobczak, Bartosz Mazur, Maura Malinska, Filip Formalik, Volodymyr Bon, Azat Khadiev, Stefan Kaskel, Bogdan Kuchta, Agnieszka M Janiak, Kornel Roztocki

**Affiliations:** † Faculty of Chemistry, 49562 Adam Mickiewicz University, Uniwersytetu Poznańskiego 8, 61-614 Poznań, Poland; ‡ Faculty of Chemistry, 49567 Wrocław University of Science and Technology, C. K. Norwida 4/6, 50-375 Wrocław, Poland; § Faculty of Chemistry, 49605 University of Warsaw, Pasteura 1, 02-093 Warsaw, Poland; ∥ Department of Chemical and Biological Engineering, Northwestern University, Evanston, Illinois 60208, United States; ⊥ Chair of Inorganic Chemistry, 9169 Technische Universität Dresden, Bergstrasse 66, 01062 Dresden, Germany; # 28332 Deutsches Elektronen-Synchrotron DESY, Notkestr. 85, 22607 Hamburg, Germany

## Abstract

Flexible metal–organic frameworks (MOFs) are porous materials exhibiting spatiotemporal responses to environmental changes, which significantly affect their sorption properties and offer potential technological breakthroughs. In this study, we investigate two isostructural thiazolo­[5,4-*d*]­thiazolate MOFs, UAM-1S and UAM-1O, which differ by a single atom, sulfur or oxygen, in the angular dicarboxylate group. Despite this subtle modification, the materials trigger distinct structural adaptation mechanisms: a continuous in UAM-1S and a discrete in UAM-1O. Using a combination of experimental and theoretical approaches, including microcrystal electron diffraction and DFT analysis, we reveal the factors driving different transition mechanism. Appropriate treatment of UAM-1O, combined with single-crystal X-ray diffraction analysis, revealed the structure of the explosive metastable open phase, corroborating theoretical predictions. Furthermore, a time-resolved *in situ* powder X-ray diffraction data set was collected under varying CO_2_ at pressures exceeding the cp-op structural transition pressure at 195 K, enabling the application of the Kolmogorov–Johnson–Mehl–Avrami equation to analyze the kinetics of adsorption. Holistically, our work enhances the understanding of the key factors responsible for the time-dependent response of flexible materials with implications for the design of dynamic materials.

## Graphical abstract

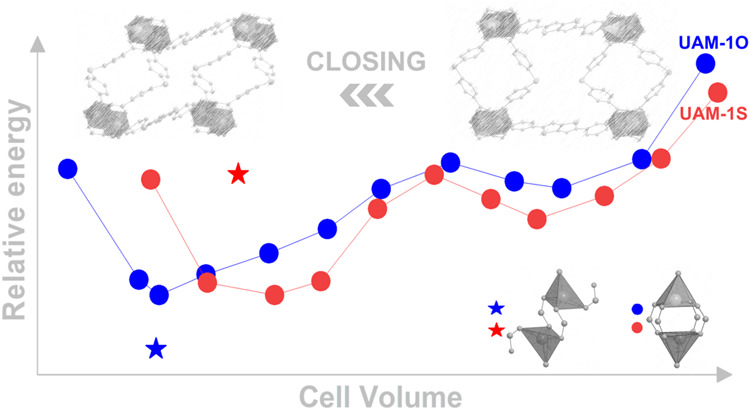

## Introduction

Growing demands for high purity chemicals,^1^ decarbonisation^2^ and fresh water^3^ put pressure on materials scientists to develop novel solid adsorbents that could lead to breakthroughs in separation and purification, water harvesting and CO_2_ capture. These materials must not only exhibit good total capacity and selectivity for a specific gas from a mixture but also ensure that the sorption/desorption cycle is fast and that as much gas as possible is excluded from the solid during the desorption process.

Recently, metal–organic frameworks (MOFs), porous coordination polymers, have emerged as solid sorbents with the potential to help resolve these pressing issues.^4–7^ The scientific community claims that they even have ability to change the chemistry of the planet.^8^ However, this potential cannot be fully unlocked without their programmable porosity based on the well-established rules of reticular chemistry,^9^ wherein the self-assembly of inorganic and organic building blocks creates repeatable voids with desirable geometries and properties. Beyond the thousands of classical “rigid” MOFs, there exists a smaller subgroup, numbering less than a few hundred, that changes structure stimulated by variations in temperature, pressure, or the presence of guest molecules.^10^ Pillared layer MOFs have been extensively studied as model systems to analyze factors affecting switchability as well as to establish advanced analytical tools and simulation methods for dynamic frameworks.^11^

The porous structure of most flexible MOFs tends to shrink during the desolvation process, resulting in a structure that is either less porous or nonporous. The accommodation of guest molecules can reverse this effect and lead to unexpected phenomena^12,13^ and peculiar isotherm shapes.^14^ Usually, these processes occur in a stepwise manner (1st order structural transition),^10^ however, there are a few MOFs that exhibit continuous changes (2nd order transition) in response to desolvation or adsorption.^15–18^

Their spatiotemporal adaptability, comparable to the induced fit model found in enzymes,^19^ opens up unprecedented horizons for applications and has been successfully employed for demanding separation, including propane/propylene,^20^ water isotopologues^21^ and hydrogen isotopes^22^ as well as for ternary gas sieving.^23^ Inspiring studies by the Zaworotko group have clearly demonstrated how flexibility can enhance the kinetics of water adsorption/desorption. In the first stage,^24^ the authors investigated rigid MOFs and showed that the kinetics of this process strongly depend on the position of the inflection points of the isotherms. Materials such as MIL-160, where the inflection point is close to RH 2–5%, show fast adsorption; however, the desorption is very slow. In contrast, MOF-303 where the inflection point is close to RH = 10%, shows moderately fast adsorption and desorption. Meanwhile, a high inflection point above RH = 20% is responsible for slow adsorption and quick desorption. They then used flexible MOFs to demonstrate that structural changes mitigate the previously observed effects, allowing both adsorption and desorption to occur quickly.^25^

Scientists have gained a good understanding of the factors responsible for framework motion during stimuli-induced structural transformations,^10,26^ including particle size,^27^ postsynthetic modification,^15,16^ and linker modification.^28^ Thus, the focus is now shifting toward the fourth dimension of stimuli-responsive materials: time.^29–31^ To the best of our knowledge, materials studied for their temporal spatial response exhibit only one type of flexibility, characterized by a stepwise adsorption mechanism.^32–34^ Taking this into account, we utilized the model materials UAM-1X (UAM-1 - Uniwersytet Adama Mickiewicza w Poznaniu material number 1; X denotes either O or S),^17^ along with state-of-the-art theoretical and experimental methods, to show that materials with a continuous breathing mechanism (2nd order transition) respond to stimuli faster than those with a gate mechanism (1st order structural transition). Microcrystal electron diffraction (MicroED) provided the missing narrow desolvated structures of UAM-1S and UAM-1O, which combined with DFT analysis uncovered the factors driving different adsorption mechanisms. Gentle desolvation of UAM-1O, a framework showing the first-order discontinuous transitions, allowed us to stabilize and resolve the structure of an metastable open phase, corroborating theoretical predictions. Furthermore, a time-dependent *in situ* powder X-ray diffraction experiment collected under varying overpressures of CO_2_ at 195 K enabled the assessment of pressure dependency of the rate constants and the dimensionality of transition. The presented holistic approach could be utilized to understand the spatiotemporal responses of flexible materials, allowing for a more deliberate material selection in applications in which timing is critical.

## Results and Discussion

### Experimental Insight into Structural Transformation

The studied materials^17^ are two isostructural, flexible, pillar-layered MOFs (Figure S1) based on zinc cations and the N-donor ligand 2,5-di­(pyridin-4-yl)­thiazolo­[5,4-*d*]­thiazole (TzTz), with either 4,4′-oxidibenzoate (oba^2–^) or 4,4′-thiodibenzoate (sba^2–^). After synthesis, these materials exist in a solvated open phase, with the molecular formulas Zn_2_(oba)_2_TzTz·DMF for UAM-1O­(op) and Zn_2_(sba)_2_TzTz·DMF for UAM-1S­(op). Notably, the materials differ only by one atom, sulfur or oxygen, which influences the ∠CXC bond angles at the bridging atoms in the carboxylate ligands. This difference consequently leads to slight variations in the pore geometry and significant changes in sorption properties (Figures S1–S3 and Table 1).

**Table 1:** Selected Structural Information on Investigated Phases of UAM-1X^a^

	UAM-1O(op)	UAM-1O(op)_M_	UAM-1O(cp)	UAM-1S(op)	UAM-1S(cp)
formula	Zn_2_(oba)_2_TzTz·DMF	Zn_2_(oba)_2_TzTz	Zn_2_(oba)_2_TzTz	Zn_2_(sba)_2_TzTz·DMF	Zn_2_(sba)_2_TzTz
space group	*P*2_1_/*n*	*Pbcn*	*C*2/*c*	*P*2_1_/*n*	*C*2/*c*
Zn–O [Å]	2.027–2.068	2.014–2.053	1.893–1.947	2.016–2.054	1.939–2.024
Zn–N [Å]	2.017–2.030	2.028–2.030	1.982	2.025–2.025	2.008
*l*_Zn–Zn_ [Å]	2.868	2.889	3.500	2.925	2.938
C-X-C [°]	115.2	117.3	118.1	101.4	103.2
*V*_v_ [%]	37.4	39.0	5.1	36.1	6.2
*d* [g/cm^3^]	1.387	1.089	1.654	1.440	1.545

a Probe radius 1.5 Å; *l*_Zn–Zn_ - distance between zinc atoms in cluster, *V*_v_ - theoretical pore volume, and *d* - experimental density calculated from structural data.

Desolvation performed under vacuum at 80 °C, resulted in distinct, unknown contracted phases with the formula Zn_2_(xba)_2_TzTz (UAM-1X­(cp); X denotes either O or S), as confirmed in our previous communications^17^ by powder X-ray diffraction and thermal analysis (Figure S4). The structural transformation caused the crystals to fragment (Figure S5), making them too small for single-crystal X-ray diffraction (SC-XRD), even with synchrotron radiation. As a result, microcrystal electron diffraction (MicroED; Figures 1 and S6) was employed to analyze the crystal structures of the contracted phases (Figures 2 and S7).

**Figure 1:**
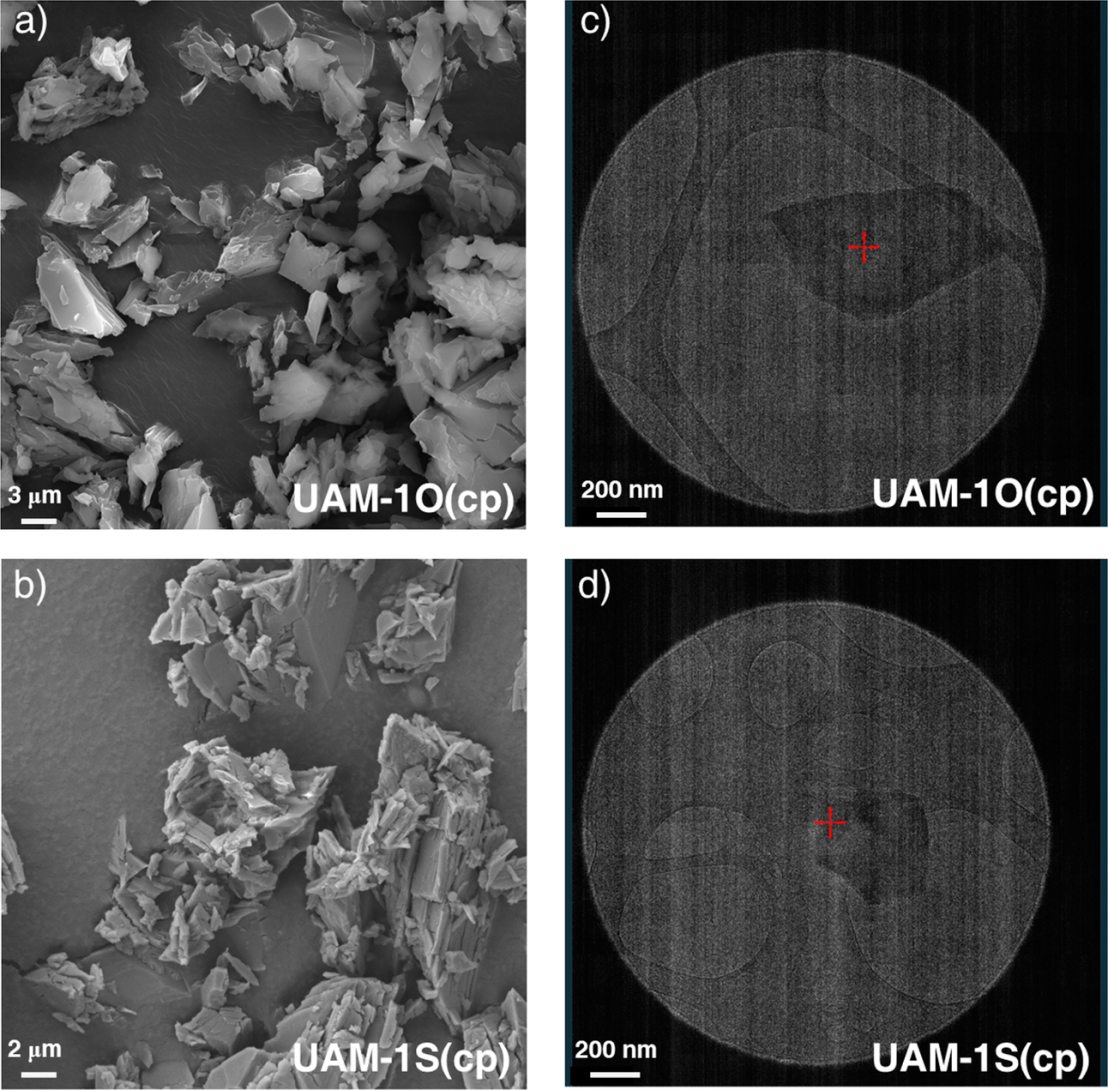
(a, b) Scanning electron microscope images of UAM-1S­(cp) and UAM-1O­(cp), along with the corresponding. (c, d) TEM images of microcrystals used for MicroED. Sample of UAM-1O­(cp), Zn_2_(oba)_2_TzTz, was prepared through four desolvation-solvation cycles of UAM-1­(op) in dichloromethane , while the sample of UAM-1S­(cp), Zn_2_(sba)_2_TzTz, was obtained through three adsorption–desorption cycles of CO_2_ at 195 K.

**Figure 2:**
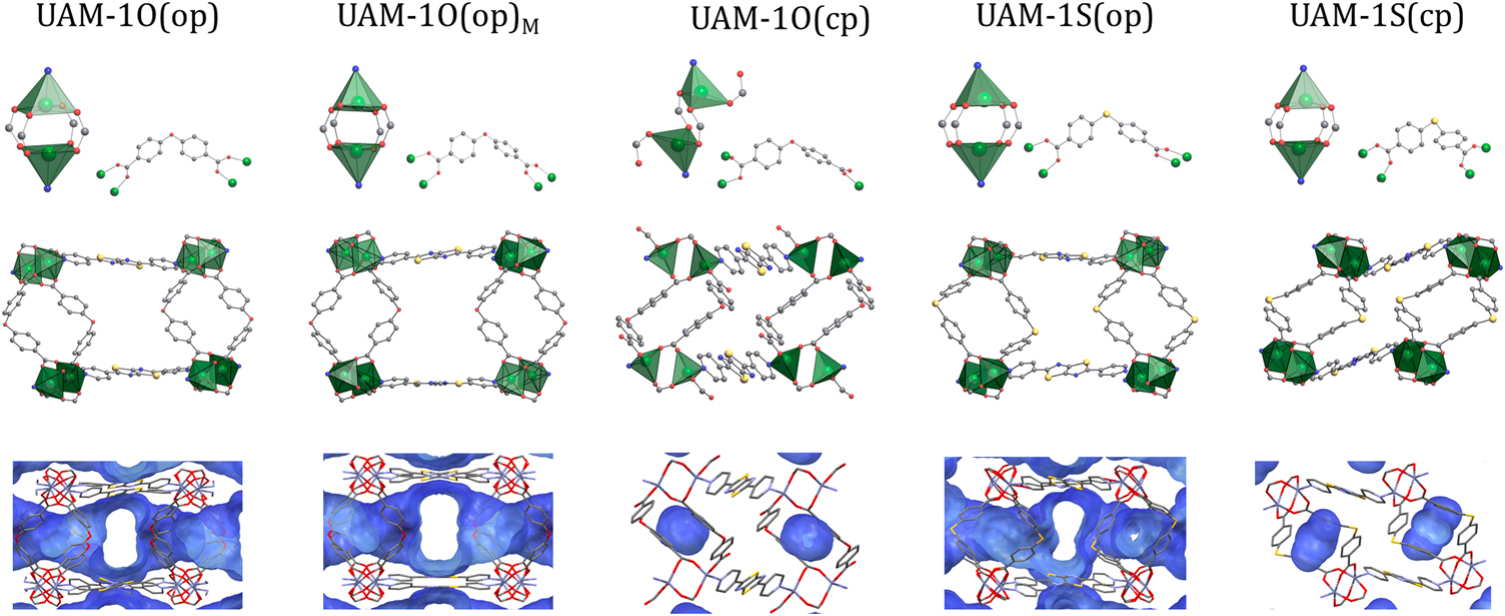
Comparison of single crystal structures of open pore phases (op) and desolvated phases (cp) of UAM-1X: metal clusters and the conformation of carboxylate ligands linked to zinc cations (top row) as well as representations of the three-dimensional frameworks with marked voids. Hydrogen atoms have been omitted for clarity; atoms are marked as follows: green - Zn, gray - C, red - O, blue - N, and yellow - S.

However, an appropriate sample preparation was required for MicroED measurements. UAM-1O underwent four desolvation/solvation cycles in DCM, while UAM-1S was treated with three CO_2_ adsorption and desorption cycles at 195 K. In both cases, “soft milling” produced a few defect-free crystals of appropriate size of 200–400 nm (Figure 1). UAM-1X­(op) crystallizes in space group *P*2_1_/*n*, but upon desolvation, symmetry changes to *C*2/*c* (Figure 2). Furthermore, this transformation is accompanied by a significant contraction, resulting in a notable decrease in the potential free accessible volume from 37.4 to 5.1% for UAM-1O, and from 36.1 to 6.2% for UAM-1S (using a probe radius of 1.5 Å; Table 1).

In the open phases, the zinc cations have a coordination number of 5, forming a paddlewheel unit in which the dicarboxylates act as μ_4_ *-κ* ^1^κ^1^κ^1^κ^1^ linkers, while the nitrogen-donor TzTz serves as a μ_2_ *-κ* ^1^κ^1^ linker. The same metal cluster geometry is also observed in UAM-1S­(cp). On the other hand, during the structural transition of UAM-1O­(op) to the closed phase, one of the Zn–O bonds in the metallic cluster breaks, leading to a reorganization of the metal cluster. Consequently, the coordination number of zinc decreases from 5 to 4, and the carboxylates shift to μ_3_ *-κ* ^1^κ^1^κ^1^ ligands. So far, similar changes in the coordination sphere have been observed in a few other MOFs.^35,36^ Breaking the bond indicates that there exists a considerable energetic barrier between the open and closed phases of UAM-1O that needs to be overcome to observe the transition. The stark density change from UAM-1O­(op) to UAM-1O­(cp) is an associated origin of barriers characteristic of first-order transitions. Theoretically, applying the right conditions to UAM-1O may result in a kinetically stabilized open phase. To investigate this hypothesis, we exchanged DMF in the pores of UAM-1O with dichloromethane multiple times. Subsequently, the sample was left at room temperature to facilitate the evaporation of DCM. While some crystals maintained their crystallinity (Figure S8), the newly formed phase, termed UAM-1O­(op)_M_, proved to be metastable. This metastability is evidenced by the heightened sensitivity of the single crystals to external stimuli, such as touch, likely caused by the sudden op-cp phase transition and the resulting powderization of the material, making them challenging to study (see the SI Movie of exploding crystals).

Such behavior was earlier observed in molecular crystals,^37–39^ but it is still rather unique for the framework materials. Nevertheless, one crystal was successfully subjected to the SC-XRD analysis. UAM-1O­(op)_M_ has the same connectivity as the corresponding UAM-1O­(op); however, it crystallizes in the orthorhombic system with the space group *Pbcn* (Table 1 and Figure S9). The void volume of this phase is calculated to be 39.0% (using a probe radius of 1.5 Å), slightly exceeding that of the original open phase, which is 37.4%. To validate the absence of guest molecules within the pores, we constructed an electron density map for UAM-1O­(op) and UAM-1O­(op)_M_ (Figure S10), and calculated the electron count within the asymmetric unit cell (ASU)^40^ for both phases. The calculations reveal that the metastable phase contains 17 electrons per formula, while UAM-1O­(op) exhibits a significantly higher electron count of 515 electrons per formula, which is 30 times greater. Seventeen electrons per formula unit correspond to 0.2 DCM molecules per zinc atom and represent 3.4% of the sample mass. Since the metastable phase is difficult to investigate and it remains unclear which phase is responsible for the abrupt structural transformation, we monitored the state of the bulk sample of UAM-1O during the following procedure: (i) DCM exchange, (ii) room temperature DCM evaporation, and (iii) mechanical perturbation (Figure 3). Comparison of the PXRD pattern of DCM-filled UAM-1O­(op)@DCMwith the calculated pattern of UAM-1O­(op)_M_ strictly indicates the similarity of these phases (Figure S8). Slow desolvation of UAM-1O­(op)@DCM leads to the expected phase mixture, containing both UAM-1O­(op)_M_ and UAM-1O­(cp) phases, as indicated by signals at 5.6° and 7.8° for UAM-1O­(op)_M_, and 9.3, 9.8, and 7. 7° for UAM-1O­(cp). Notably, the 7.7° signal from the cp phase is overlapped to 7.8 UAM-1O­(op)_M_. Subsequent mechanical perturbation by the needle (please see Movie) results in a decrease in the amount of the open phase, accompanied by an increase in the cp phase, manifested as an increase in the intensity of the 7.7 and 9.8° peak, and a corresponding decrease in the 5.6° peak.

**Figure 3:**
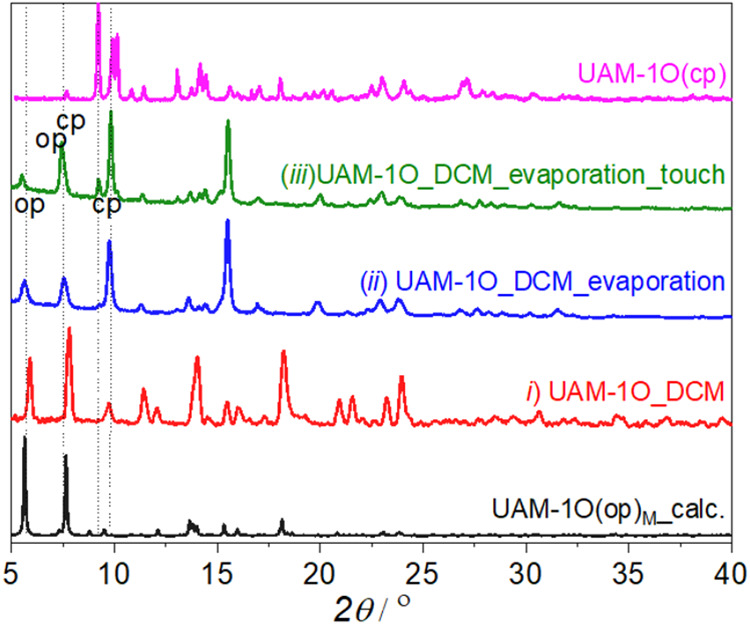
Evolution of PXRD patterns of UAM-1O through the following steps: (i) DCM exchange, (ii) room temperature DCM evaporation, and (iii) mechanical perturbation compared with the calculated patterns of UAM-1O­(op)_M_ and the experimental pattern of UAM-1­(cp). For details on the mechanical perturbation, refer to the accompanying video.

The comparison of calculated PXRD of UAM-1­(op), UAM-1­(op)_M_ and UAM-1­(cp) with the bulk sample after the DCM evaporation indication that the op phase that undergoes structural changes when touched with a needle most closely resembles UAM-1O­(op)_M_ (Figure S11), however there are some discrepancies.

Additionally, we investigated the bulk sample after the evaporation of DCM under ambient conditions by thermogravimetric analysis (Figure S12), monitoring the ion signals corresponding to water (*m*/*z* = 18) and DCM (*m*/*z* = 84). Prior to reaching the long plateau between 160 and 290 °C, the sample lost approximately 3.5% of its mass. The *m*/*z* = 18 signal reaching a maximum at 55 °C, indicating the release of water molecules adsorbed on the crystal surface. Additionally, around 35 °C, the sample began to lose DCM, which was clearly detected by a rising *m*/*z* = 84 signal. The signal from DCM reaches a maximum at approximately 98 °C. Those DCM molecules represent the observed electron density in SC-XRD analysis of UAM-1O­(op)_M_.

In the final step, we investigated the behavior of UAM-1O­(op)@DCM under a vacuum at room temperature. The sample was subjected to low pressure overnight, after which its state was monitored. The sample was fully powdered, and no distinct single crystals were observed. PXRD analysis revealed a mixture of phases, dominated by the cp phase, with only a minor amount of the open phase, which in response to touch by needle completely transforms into the closed phase, as evidenced by the disappearance of the reflection at 5.6° (Figure S13). Made observation highlights the crucial role of slow DCM evaporation at ambient conditions.

This allows us, with high probability, to postulate that the UAM-1O­(op)_M_, containing minor amounts of ordered DCM and water on the surface, is responsible for the observed explosion, following the pathway: UAM-1O­(op)_M_ → UAM-1O­(cp). Interestingly, the theoretical insight provided in the subsequent section predicts the existence of a transient intermediate phase with higher symmetry before transitioning to the closed phase and the structural transformation visualization proved the existence of the kinetically trapped phase (please see UAM-1O_animation).

On the other hand, regardless of how UAM-1S­(op) is desolvated, only a closed phase can be obtained. Notably, altering the solvent from DMF to DCM had a pronounced impact on the sample, leading to immediate cracking of the crystals. A similar effect is observed when the UAM-1S­(op) Zn_2_(sba)_2_TzTz·DMF sample is exposed to ambient conditions. Such a prominent difference in the desolvation behavior represents a clear experimental evidence of discontinuous in UAM-1­(O) and continuous UAM-1­(S) guest-induced structural transitions.

### Theoretical Insight into Structural Transformation

The experimental observations indicate a considerable difference in the manner in which UAM-1S and UAM-1O respond to the desolvation process. Upon closure, UAM-1O exhibits a structural rearrangement in the Zn-node, whereas UAM-1S undergoes closure without rearranging its Zn–O bonds. In order to provide theoretical insight into this behavior, a series of periodic DFT calculations was conducted to compare the energy-volume profile of both materials (for full description of computational techniques, see SI). In the initial step, we constructed a closed pore configuration with a preserved paddlewheel (cp_1_) for both UAM-1 variants by optimizing an experimentally derived open phase under constant pressure. By interpolating the volume and atom positions between two phases, a set of structures with different volumes was created. These structures were then reoptimized at constant volume (with optimization of the atom positions and the cell vectors), resulting in the generation of energy-volume profiles (Figure 4a). Two energy minima were identified for both UAM-1 variants, corresponding to the open and closed phases. It is noteworthy that the closed-phase configurations of both materials are more stable than their open-phase counterparts, which is consistent with experimental observations of self-closing behavior following desolvation.

**Figure 4:**
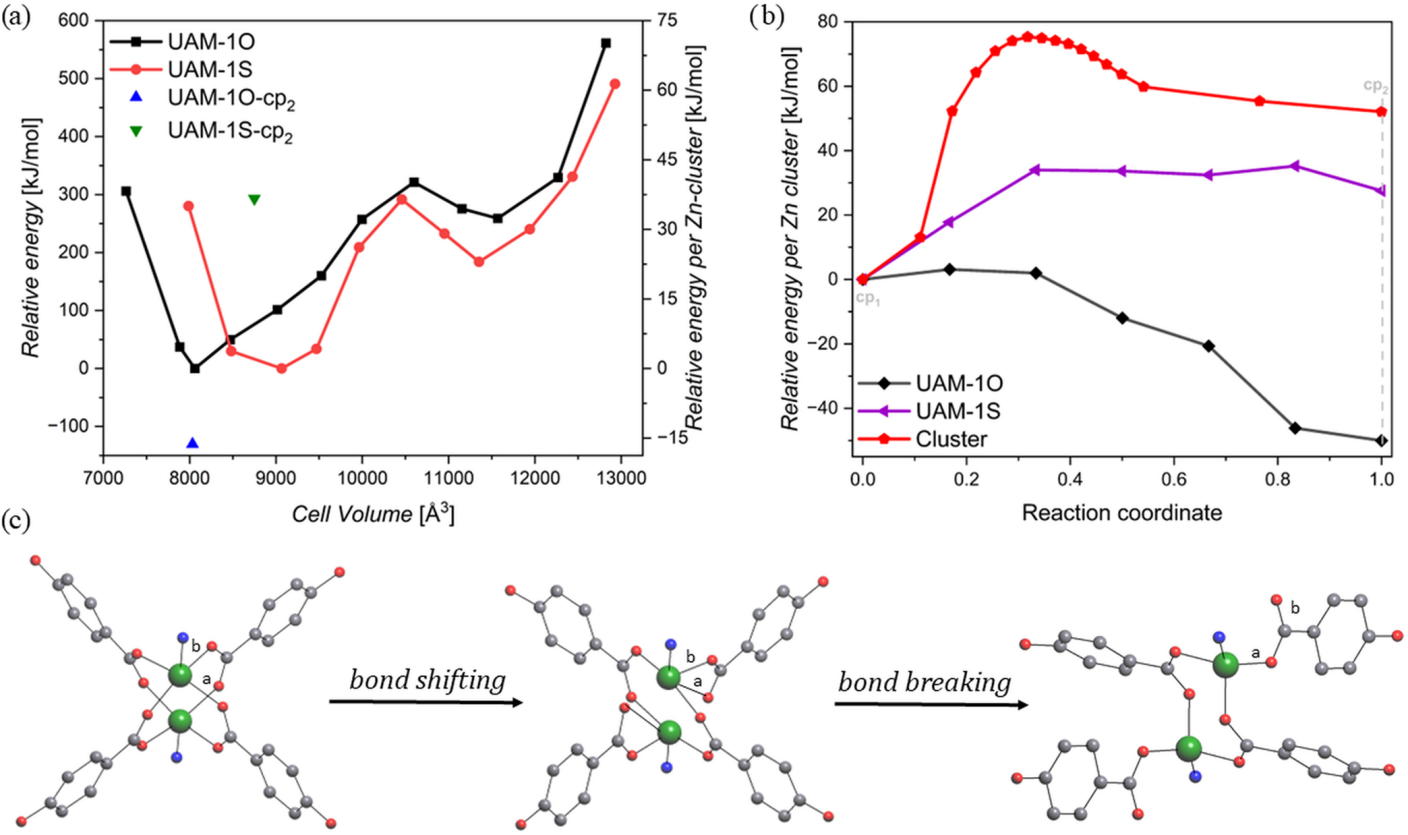
(a) Relative electronic energy. The values for both materials were calculated independently by subtracting from all values the value of minimum energy for a given cp_1_ variant, without Zn–O bond breakage. (b) Energy profile calculated per single Zn-node for the transformation of the cp_1_ to cp_2_ configuration. Data for UAM-1O (Zn_2_(oba)_2_TzTz) and UAM-1S (Zn_2_(sba)_2_TzTz) were calculated using solid-state NEB, while data for cluster (Zn_2_O_4_N_2_) was calculated using NEB. NEB = nudged elastic band methodology. (c) Schematic representation of the considered transition mechanism. The initial structure cp_1_ is presented on the left with two oxygens labeled as a and b, in the middle, the transition path with breaking both Zn–O­(b) bond and the formation of new Zn–O­(a) bond, and on the right are the resulting structures with different resulting nonbonded oxygens. Please note that cp_1_ denotes the contracted phase of UAM-1S or UAM-1O, where the coordination number of zinc and the paddlewheel are preserved, while cp_2_ refers to contracted phases with a decreed zinc coordination number to 4 and a disassembled paddlewheel. All calculations, except for cluster, were done for unit cell containing 16 Zn atoms.

In UAM-1O, however, an additional bond rearrangement was observed in the closed phase during the experiment. Although this rearranged closed pore configuration (denoted as cp_2_) was not directly reproduced during standard computational optimization of the cp_1_ structure, it was obtained by optimization of the experimentally derived structure. As the cp_2_ structure of UAM-1S has not been observed experimentally, we created it by modifying the UAM-1O structure. The results demonstrate that the bond rearrangement in UAM-1O (cp_1_ → cp_2_) yields a more stable closed pore configuration, whereas a similar rearrangement in UAM-1S leads to a configuration less favorable in energy. This is consistent with experimental observation, where no rearrangement in the Zn-node was observed in UAM-1S during closing.

In order to gain a deeper understanding of the underlying mechanism of the bond rearrangement in UAM-1O, we applied solid-state nudged elastic band (ssNEB) calculations,^41^ as well as NEB calculations on a Zn-node cluster model (Figure 4b). As both methodologies require a set of initial configurations, two mechanisms were considered: (i) the breaking of a single Zn–O bond and the rotation of the COO group and (ii) the breaking of both Zn–O bonds, the shift of the COO group, and the creation of a single Zn–O bond (Figures 4c and S15). As the rotation variant did not achieve a reasonable convergence, it can be concluded that the shifting mechanism is responsible for the transition. Consequently, only this variant is studied further.

In accordance with the preceding results, a reduction in energy is evident during the transformation of UAM-1O from the cp_1_ to the cp_2_ configuration. Conversely, a comparable transformation of UAM-1S results in an increase in the total energy of the system. In both cases, no energy barrier was observed between the two states. However, this apparent lack of an energy barrier may be attributed to compensatory effects, such as the reduction of lattice or bond strain during the transition, which offset the energetic cost of bond breaking and reformation. To test this hypothesis, we constructed a cluster model of the Zn-node in which we performed an analogous cp_1_ to cp_2_ shift transition (for a detailed description, see SI). This transition resulted in both a higher-energy structure and an energy barrier between states of 75.3 kJ/mol per Zn-node (Figure 4b). Therefore, it can be concluded that the rearrangement is indeed a highly energetically demanding process, though the crystal environment is likely to influence the effective barrier.

The remaining issue is the observation of the metastable UAM-1O phase, which is not a direct product of cp_1_ to cp_2_ rearrangement. An alternative hypothesis is that the metastable UAM-1O phase may result from a distinct lattice geometry (*Pbcn*) that the crystal exhibits as it evolves between open (*C2*/*c*) and closed configurations (*P*2_1_/*n*). In UAM-1O, the unit cell angles undergo a notable evolution (from β ∼ 84° in the op phase to β ∼ 90° in the metastable phase, and then to β ∼ 124° in the cp phase), resulting in a strained intermediate state that fails to fully relieve internal stresses. This strained geometry may be indicative of a kinetic trap, resulting in the emergence of a metastable phase that is mechanically fragile and prone to collapse. In contrast, such a metastable state is not observed for UAM-1S. Although the op structure also deviates from right angles (β ∼ 87.4°), the subsequent transition to the cp phase results in a substantially different angle (β ∼ 56.4°) without passing through a near-orthogonal state. Consequently, UAM-1S does not undergo the same pronounced progression of lattice angles, thereby avoiding the formation of a similarly trapped metastable configuration.

In a previous study,^17^ we demonstrated that UAM-1S and UAM-1O exhibited a unique dynamic response to CO_2_ at 195 K (see Figure S3). In-situ techniques, including PXRD and time-resolved PXRD, were employed to identify the adsorption mechanisms and determine the underlying mechanisms. In the case of UAM-1O, the adsorption mechanism was identified as gating, while in the case of UAM-1S, it was determined to be continuous breathing. In light of the aforementioned findings, it can be stated with certainty that the stabilization of the closed pore phase of UAM-1O with the broken bond is responsible for the gating mechanism, while the lack of reorganization of the cluster drives the continuous breathing of UAM-1S in response to CO_2_.

While the aforementioned statements are true in their nature, they do not provide an answer to the following question: how does a single atom change influence the observed spatiotemporal response? Taking this into account, we have revisited both experimental and theoretical structures, including the open and closed phases of UAM-1O and UAM-1S. After careful deliberation, we focused on three key factors: the C–X–C angle, the C–X bond length, and the planarity of the sba^2–^ linker benzene ring, where X stands for O or S. Planarity is defined as the measure of the deviation of individual atoms of a ring from the plane to which they are fitted (for a full definition, see SI), where a value of 0 indicates a perfectly flat ring.

A visual comparison of the oba^2–^ and sba^2–^ linkers extracted from periodic structures of different variants of UAM-1 materials (Figure 5) suggested distinct responses to structural compression during the transition to the closed phase. Based on the experimentally recorded UAM-1O closed phase, open phase, metastable (meta), as well as UAM-1S­(cp) and op and UAM-1O­(op)­theoretically derived structures, we conducted a structural analysis of the linkers (Figures 5 and S16), calculating angles between selected carbon atoms and the bridging atoms (oxygen or sulfur), and assessing the planarity of the carbon atoms of the aromatic rings and carboxyl groups.

**Figure 5:**
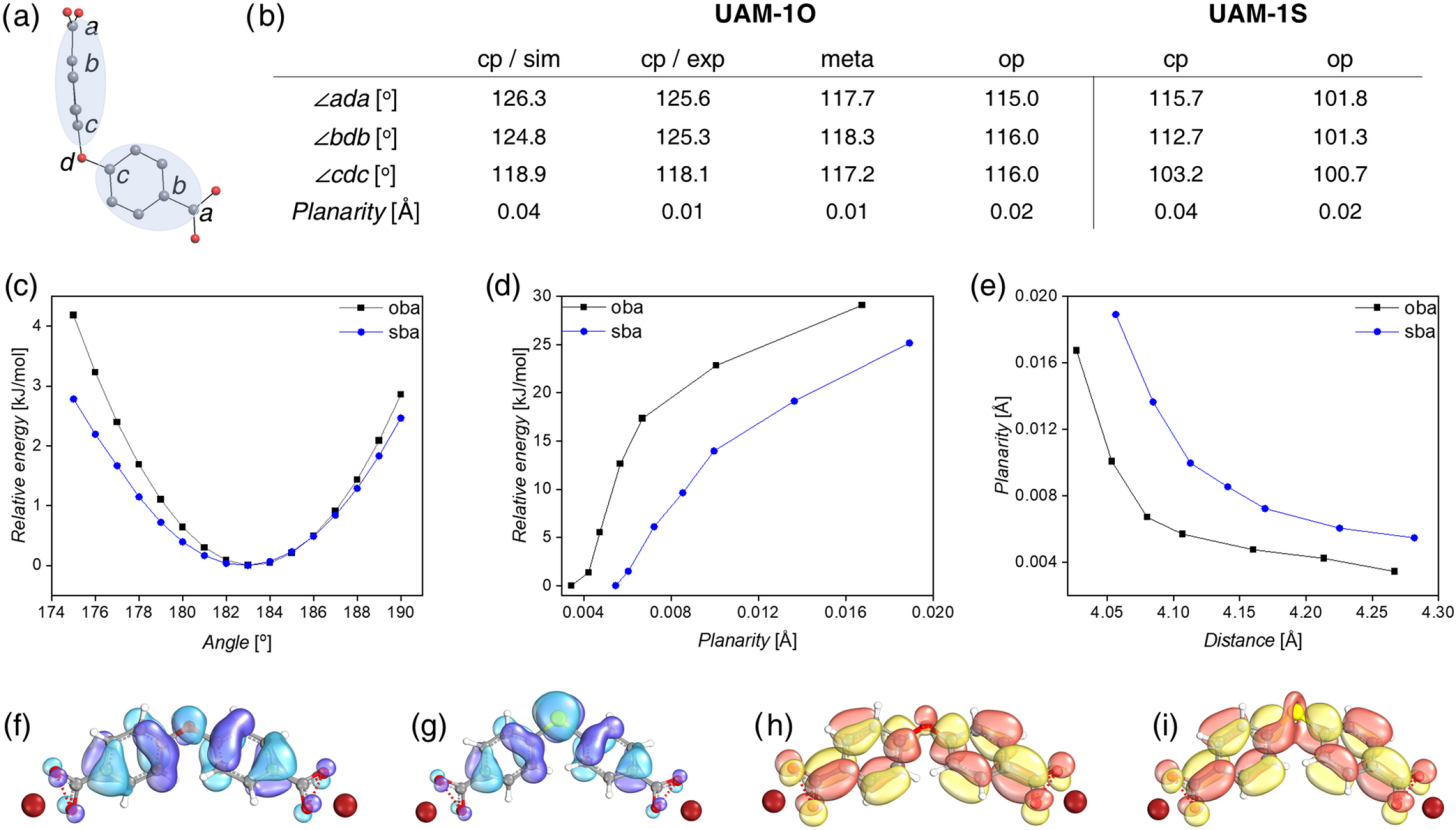
(a) Representation of linker extracted from periodic UAM-1O­(op). (b) Structural data calculated on fragments extracted from periodic UAM-1X structures (Figure S13). All data calculated is as an average value based on 16 extracted linkers, except for experimental UAM-1O­(cp) and UAM-1S­(op), which were calculated on 8 linkers. Energy profiles illustrate: (c) energy change as a function of the out-of-plane angle, (d) energy change as a function of benzene ring planarity, and (e) the relationship between benzene ring planarity and the distance between the carbon atom connected to the bridging atom (O or S) and the carboxyl group carbon, for both oba^2–^ and sba^2–^ linkers. Visualization of HOMO orbitals for (f) oba^2–^ and (g) sba^2–^ linkers compared with corresponding LUMO orbitals for (h) oba^2–^ and (i) sba^2–^ linkers. For more details, please see Figures S15 and S16.

A key observation is that the benzene ring of the sba^2–^ linker is notably less planar than that of the oba^2–^ linker. Experimental data from the closed phase confirm this difference, with planarity values of 0.01 Å for oba^2–^ and 0.04 Å for sba^2–^ benzene rings. Importantly, the DFT-optimized structure of UAM-1O­(cp) with intact bonds also exhibited a benzene ring planarity of 0.04 Å, similar to that of sba^2–^ in the closed phase (Figure 5b). This observation suggests that geometric constraints during structural closure induce bending of the linker. Due to oxygen’s lower polarizability, higher electronegativity, and smaller atomic radius compared to sulfur, the C–O–C linkage in oba^2–^ is inherently shorter and stiffer than the C–S–C linkage in sba^2–^. Consequently, sba^2–^ is more deformable and can adjust its geometry more effectively within the closed MOF structure without a significant rearrangement of bonds to the zinc nodes.

To verify these structural observations, we performed DFT calculations assessing the flexibility of dicarboxylic linkers. Initially, oba^2–^ and sba^2–^ linkers were extracted from the MOFs, capped with Li^+^ ions to maintain neutrality, and optimized at the r2SCAN-3c/def2-mTZVPP level of theory.^42^ Following optimization, structures were systematically modified by adjusting the out-of-plane angle of the bridging atom (oxygen or sulfur) within a range of 175–190°, with a fixed X–C bond length (Figure S17). The angle scan resulted with the lowest energy for an angle of 183° for both linkers (we note that this is the point with the lowest energy from the scan, not a value from a fitted parabola), consistent with experimental data, while clearly illustrating higher stiffness in oba^2–^ (Figure 5c). Additionally, linker flexibility under compression was analyzed by varying the distance between aromatic ring carbons and carboxyl group carbons within specific ranges (4.000–4.267 Å for oba^2–^, 4.000–4.282 Å for sba^2–^) and further reoptimization of remaining atoms. We employed this methodology to investigate the way aromatic ring behaves when it is squeezed (as during the phase transition). For the optimized structures, the planarity of the resulting aromatic rings was calculated using the previously introduced methodology. The energy-planarity profiles (Figure 5d,e) clearly revealed that oba^2–^ rings resist deformation more strongly than sba^2–^, with sharper increases in energy upon deformation and a more planar minimum-energy conformation.

Finally, to elucidate the electronic origin of these mechanical differences, HOMO/LUMO orbital analyses were conducted (Figure 5). The calculated HOMO–LUMO energy gaps revealed a smaller gap for sba^2–^ (3.26 eV) compared to that for oba^2–^ (3.83 eV). Furthermore, the orbital visualizations (Figure 5) highlighted substantial differences: sba^2–^ exhibited continuous LUMO (Figures 5i and S19) orbital delocalization across the bridging sulfur, facilitating electronic rearrangement and flexibility. In contrast, oba^2–^ displayed discontinuous LUMO orbital distributions near the oxygen bridge (Figures 5h and S19), significantly limiting electronic delocalization. If the LUMO is delocalized over atoms involved in a bending mode (such as a three-atom angle), it may indicate that upon excitation, or under external stress, the system could redistribute electron density in a way that stabilizes deformation. This further substantiates the hypothesis that the sba^2–^ linker facilitates enhanced mechanical deformability, while the oba^2–^ linker favors rigidity, resulting in phase transition involving Zn–O bond rearrangement.

### Kinetic Studies of CO_2_-Induced Structural Transition

Herien we utilized time-resolved *in situ* PXRD to monitor the kinetics of structural transitions (cp → op) of UAM-1O and UAM-1S induced by carbon dioxide at varying pressure jump conditions (from vacuum to 30–60 kPa) of CO_2_ at 195 K (Figure 6). Before the final experiments, the samples were soaked in DCM four times, then evacuated at 80 °C for 8–14 h, and additionally exposed to three CO_2_ adsorption–desorption cycles. As in previous reports,^32,43^ Kolmogorov–Johnson–Mehl–Avrami (KJMA)^44^ equation was employed in the mathematical description of observed structural transitions. The equation takes the form *a* = 1 – exp­(−*kt* ^*n*^), where *a* represents the fraction of the open phase, *k* is the rate constant, *t* is the time, and *n* is the dimension of the structural transition. During the experiment, we observed two key phenomena: first, the system exhibited a delayed gas adsorption response that varied depending on the sample and the applied pressure, pointing out the kinetic barriers of cp → op transitions. Therefore, all analyses were conducted starting from the so-called induction time, *t*_0_, indicating the onset of the structural transition from the closed phase to the open phase (Table 2). Second, as time progressed and the structural transition was fully completed, a gradual decline in the fraction of the open phase was observed. This was attributed to the destructive effect of high-intensity X-ray radiation on the sample, which manifested as black spots (Figure S20).

**Table 2:** Experimental and Calculated Parameters for *In Situ* Synchrotron PXRD Measurements on UAM-1O and UAM-1S^a^

UAM-1O						UAM-1S					
*p* [kPa]	*t*_0_ [s]	*t*_e_ [s]	*n*	*k* [*s* ^*1*/*n*^]	*R* ^2^	*p* [kPa]	*t*_0_ [s]	*t*_e_ [s]	*n*	*k* [*s* ^*1*/*n*^]	*R* ^2^
60	4.3	50	1.12	0.031	0.989	60	4.1	19.8	1.43	0.076	0.986
50	5.7	140	0.91	0.030	0.992	50	5.6	29.4	1.38	0.028	0.976
40	6.3	150	0.95	0.023	0.994	40	9.3	39.0	1.17	0.042	0.993
30	8.1	250	0.83	0.025	0.978						

a *p* - pressure, *t*_s_ - induction time, *t*_e_ - structural transition end time, *n* - dimension of the structural transition, and *k* - rate constant.

According to the previous work on the crystallization process of alloys,^45^ the relation between ln­[−ln­(1 – *a*)] and ln­(*t*) was used to find *n*, where *a* is the fraction of the open phase. In the case of UAM-1O, the exponent *n* changed with pressure, e.g., *n* is equal to 1.12 for 60 kPa while *n* is equal to 0.83 for 30 kPa (Figures 6, S21 and Table 2). Furthermore, at 60 kPa, the time between gas inlet time (*t*_s_) and adsorption initiation time (*t*_0_) is 4.3 s, and the structural transition end time (*t*_e_), defined as maximum fraction of the open phase, is reached after approximately 25 s. At 30 kPa, the time between *t*_s_ and *t*_0_ is 8.1 s, with the maximum fraction of the open phase appearing after approximately 240 s. Due to the different units of the rate constant, equal to s^1/*n*^ (s -second; *n* - the dimension of the structural transition), they cannot be directly compared.

**Figure 6:**
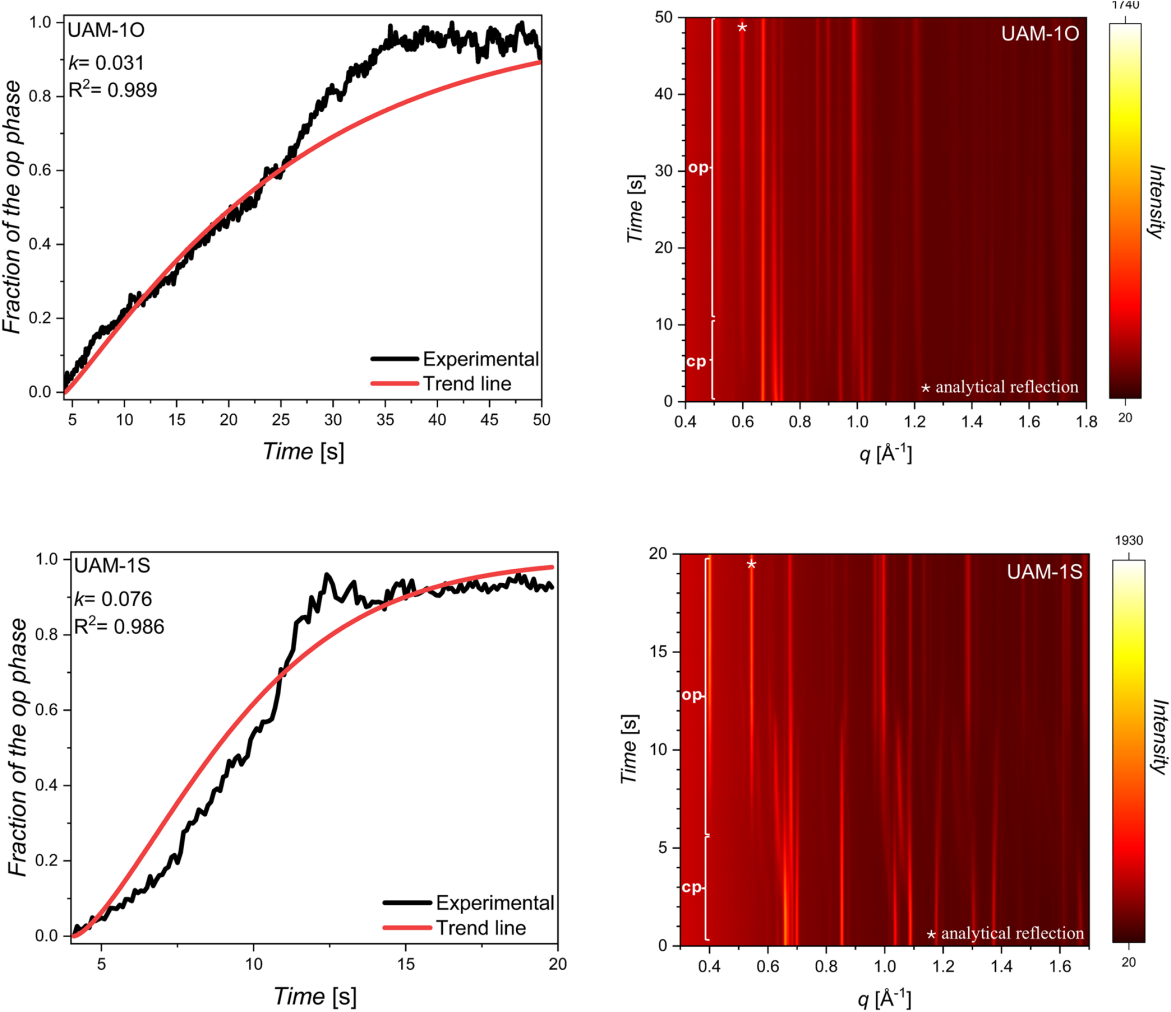
Fractions (left) of the open pore phase for UAM-1X calculated from *in situ* time-dependent PXRD measurement (right) upon CO_2_ adsorption at 60 kPa. The normalized peak intensity and trend line were obtained by fitting the experimental data to the KJMA equation, *a* = 1 – exp­(−*kt^n^*).

On the other hand, UAM-1S exhibits a mechanism described as continuous breathing. For this framework, the exponent varied from *n* = 1.43 (60 kPa) to *n* = 1.17 (30 kPa). Similar to the case for UAM-1O, the rate constants cannot be directly compared. However, based on the transformation time, we can conclude that the phase transformation for UAM-1S occurs faster than for UAM-1O. For instance at 60 kPa, the complete structural transition takes approximately 10 s, making this process 15 s faster than for UAM-1O. Qualitatively spoken, the larger volume change and bond reformation in the UAM-1O transformation signify a higher activation energy leading to a first-order transition, while the modest cell volume change in UAM-1S leads to a quasi-continuous transformation with significantly reduced activation barrier and accelerated response function.

So far, only a few materials have been studied using time-resolved PXRD along with analysis based on the KJMA equation. For ELM-11,^33^ the exponent *n* in all studies fell within the range of 1.2–1.3, indicating a quasi-one-dimensional growth of the open phase, which reflects a gating mechanism. In the case of DUT-8­(Ni), the structural transition induced by DCM vapor adsorption is described as gating,^32^ with an *n* value of 1.31. Substituting part of the nickel ions with cobalt ions alters the exponent *n*, e.g., for DUT-8­(Ni_0.75_Co_0.25_) is 1.51. The rate constants for the structural transition also change with *k* = 0.238 for DUT-8­(Ni) and *k* = 0.104 for DUT-8­(Ni_0.75_Co_0.25_).

At each pressure, a consistently higher value of the phase transition dimension (*n*) is observed for UAM-1S compared to that for UAM-1O. We attribute this to the underlying adsorption mechanism. In the case of UAM-1O, the gating process, driven by bond rearrangement, allows only two discrete structural states—open and closed—there by limiting its structural flexibility. In contrast, the preservation of cluster connectivity, combined with the greater deformability of the sba^–2^ linker—as demonstrated by DFT analysis—enables significantly higher dimensionality of structural adaptation in UAM-1S. This is reflected in its considerably higher *n* values at each evaluated pressure (Table 2).

The fitted KMJA model represents one of the possible approaches that can be applied to the experimental data. Another interesting example is the method employed by Watanabe and co-workers,^43^ who monitored the phase transition of ELM-11, MIL-53, and CuFB during a steady pressure increase using time-resolved *in situ* X-ray powder diffraction. This approach allowed them to plot the fraction of the open phase versus pressure and assign distinct kinetic models to each framework. ELM-11 exhibited an autocatalytic transition, and MIL-53 followed a first-order reaction, while CuFB was best described by a zero-order reaction model.

We applied a similar approach to the investigated samples of UAM-1S and UAM-1O under three different pressure rates (ν_t_): 0.2, 0.4, and 0.8 kPa·s^–1^ (Figures S23 and S24). For UAM-1O, we calculated ν_t_·(dα/dP) values for different α values ranging from 0.1 to 0.9. This was expected to allow the determination of the slope and *x*-intercept of the corresponding functions. However, we did not obtain satisfactory linearity, as shown in Figure S25b. This is a consequence of the unique CO_2_ adsorption mechanism of the UAM-1X materials.

In the first step, approximately 20 cm^3^·g^–1^ CO_2_ diffuses into the zero-dimensional voids of UAM-1X frameworks (Figure S3), which practically does not change their structure.^17^ Subsequently, a structural transition has happened. This behavior differs from that of ELM-11, MIL-53, and CuFB, where no intermediate gas-filled state exists prior to the structural transition. The two-step mechanism investigated in UAM-1S and UAM-1O heavily depends on the diffusion of CO_2_ into the 0D cavities, which is strictly connected with the crystal size. As shown in the SEM images and also observed in the diffraction images from time-resolved studies, the crystal size distribution is not homogeneous (Figure S26), which plays a crucial role in the fluctuations of the intensity of reflections, in particular reflections belonging to the op phase of UAM-1S, during the semiequilibrated experiments with steadily increasing pressure. This indicates the limitation of the proposed approach to the samples with small crystals and homogeneous crystal size distribution, assuring reliable statistics in PXRD patterns and similar induction time.

## Conclusions

Herein, through a well-designed sample preparation process for MicroED measurements, we overcome the challenges posed by fragmentation of flexible MOF crystals during desolvation. As a result, we determined the previously unknown crystal structures of the closed phases UAM-1O­(cp) and UAM-1S­(cp). Although the open phases are isostructural, the closed phases exhibit significant differences, particularly in the structure of the metal cluster. For UAM-1S­(cp), the paddlewheel structure is preserved, while desolvation of UAM-1O breaks the zinc–oxygen bond, reducing the coordination number of zinc from five to four.

Density functional theory and solid-state nudged elastic band computational experiments were used to achieve a detailed understanding of the structural transition energetics. Furthermore, we determine the precise mechanism of cluster disassembly and reassembly in UAM-1O, including the energy of this process. On the other hand, calculations assessing the flexibility of dicarboxylic linkers indicate that the sba^2–^ linker facilitates enhanced mechanical deformability, while the oba^2–^ linker promotes rigidity, resulting in phase transition involving Zn–O bond rearrangement.

The existence of a kinetically stabilized UAM-1­(op)­M confirms that the reconfiguration of the zinc cluster proceeds through an orthogonal state, implying a gated mechanism. To the best of our knowledge, this metastable phase is the first example of an exploding MOF crystal, which in its nature resembles molecular crystals.

Additionally, time-resolved PXRD studies conducted during CO_2_ adsorption at 195 K, in combination with the application of the KJMA equation, revealed distinct characteristics among the UAM-1X phases. These studies demonstrate that the dimensionality of the structural transition (*n*) depends on both the system and the pressure, which implies that the rate constant (*k*) is not directly comparable across different systems. Taking this into account, we suggest that the structural transition end time (*t*_e_) at a defined threshold pressure and temperature could serve as an indicator for comparing the responses of different flexible MOFs toward the same gas.

In summary, we combined experimental and theoretical approaches for understanding dynamic MOFs in the time domain. Our studies demonstrate that flexible MOFs, which undergo continuous structural changes in response to external stimuli, exhibit faster and more energy-efficient transitions. These properties potentially enhance their applicability in gas storage, separation, and purification.

## Supplementary Material









## Notes

UAM-1O_animation (MP4)
UAM-1S_animation (MP4)
UAM-1O_explosion (MP4)
PXRD patterns, crystal structure drawings, synthetic procedures, crystallographic tables, crystal images, and additional experimental and theoretical procedures (PDF)
