# Supplementary material for: How a Single Atom Influences the Spatiotemporal Response of Flexible MOFs: Insights from Theory and Experiment
Source: J Am Chem Soc. 2025 Jun 16;147(25):21575–85. doi: 10.1021/jacs.5c02552 (PMC12203614; doi:10.1021/jacs.5c02552)
Supplement: Supplementary file 4 [file ja5c02552_si_004.pdf]

Supporting Information

How a Single Atom Influences the Spatiotemporal Response of Flexible MOFs: Insights from Theory and Experiment

Szymon K Sobczak[a], Bartosz Mazur[b], Maura Malinska[c], Filip Formalik[d], Volodymyr Bon[e], Azat Khadiev[f], Stefan Kaskel[e], Bogdan Kuchta[b], Agnieszka M Janiak[a], Kornel Roztocki*[a]

[a]*Faculty of Chemistry, Adam Mickiewicz University, Uniwersytetu Poznańskiego 8, 61-614 Poznań, Poland*
[b]*Faculty of Chemistry, Wrocław University of Science and Technology, C. K. Norwida 4/6, 50-375Wrocław, Poland*
[c] *Faculty of Chemistry, , University of Warsaw, Pasteura 1, 02-093 Warsaw, Poland*
[d] *Department of Chemical and Biological Engineering, Northwestern University, Evanston, Illinois 60208, United States*
[e] *Chair of Inorganic Chemistry, Technische Universität Dresden, Bergstrasse 66, 01062 Dresden, Germany*
[f] *Deutsches Elektronen-Synchrotron DESY, Notkestr. 85, 22607 Hamburg, Germany*

Table of Contents

Experimental and Theoretical Procedures 3

Figures 6

Tables 24

Literature 26

Figure S1 Pore geometry (a and b) and carboxylate ligand along with the C-X-C angle (X:O or S): Top row UAM-1O(op); bottom row UAM-1S(op). 6
Figure S2 Pore size distribution of different phases of UAM-1O (left) and UAM-1S (right). 7
Figure S3 Mechanistic understanding of CO_2-driven structural transformation in UAM-1X (O – top row; S – bottom row): (a) CO_2 adsorption (full symbols) and desorption (open symbols) at 195 K juxtaposed with (b) corresponding in situ PXRD patterns collected at selected pressure (λ = 1.540599 Å); (c) in situ time-resolved PXRD during the CO_2 adsorption at 195 K. m_{pd}: maximum pore diameter; p_{ws}: pore window size; V_{pt}, theoretical pore volume; and V_{pe}, experimental pore volume for CO_2 adsorption calculated at ~0.99 bar according to the Gurvich's rule. Reproduced with permission from the Royal Society of Chemistry.[28] 7
Figure S4 Powder diffractograms of open and closed phases of UAM-1X. (left) Thermal gravimetric analysis UAM-1O (red) UAM-1S (black), the chart shows the percentage of weight loss (right). 8
Figure S5 UAM-1X (X: O or S) microscopic images of crystals (with scale bar) after synthesis (left) and after activation (right; DCM exchange; 80 °C and vaccum) taken under polarized light. 8
Figure S6. (**a**) and (**b**) Scanning electron microscope images of UAM-1S(cp) and UAM-1O(cp), along with corresponding (**c**) and (**d**) TEM images of microcrystals used for microED. UAM-1O(cp) was prepared through five desolvation-solvation cycles of UAM-1(op) in DCM, while UAM-1S(cp) was obtained through adsorption-desorption cycles of CO2 at 195 K. UAM-1O(cp) = $Zn_2(oba)_2TzTz$, and UAM-1S(cp) = $Zn_2(sba)_2TzTz$. 9
Figure S7 a) UAM-1O(cp) coordination environment; b) UAM-1S(cp) coordination environment; c) 2D layer formed by $Zn(oba)_2$ and d) $Zn(sba)_2$ (projected along the [100] direction). 10
Figure S8 Microscope photos (with scale bar) of **a**) UAM-1O soaked in DCM (left) and the same crystals after evaporation of **b**) DCM in ambient conditions (right); most of the crystals have been defragmented and powdered, but there are some crystals visible that are unchanged and are likely to be UAM-1O$(op)_M$**.** **c**) Comparison of PXRD patterns of UAM-1O after soaking in DCM with the calculated patterns of UAM-1O$(op)_M$; due to fast DCM evaporation rate, the measurement was done in the closed capillary. **d**) Evolution of PXRD patterns of UAM-1O through the following steps: 1) soaking in DCM (red), 2) evaporation at ambient conditions (blue), and 3) touching (green), compared with the calculated patterns of UAM-1O$(op)_M$ (black) and the experimental pattern of UAM-1(cp), magenta. For details on the mechanical perturbation, please refer to the accompanying video. 11

Figure S9 UAM-1O$(op)_M$: a)coordination environment, b) layer composed of $Zn(oba)_2$ and d) the three-dimensional UAM-1O(op)M framework. d) Overlay of UAM-1O(op), black, with UAM-1$(op)_M$, red, structures along the z axis. 12
Figure S10 Electron density maps for UAM-1O(op) and for UAM-1O$(op)_M$. 13
Figure S11 The comparison of calculated PXRD of UAM-1(op), UAM-1(op)M and UAM-1(cp) with the bulk sample after the DCM evaporation at ambient conditions (top). The comparison of calculated UAM-1(op)M with the bulk sample after the DCM evaporation (down). 14
Figure S12 TG-MS analysis of the bulk sample after the DCM evaporation. 15
Figure S13 Comparison of the PXRD patterns of the calculated UAM-1O$(op)_M$ with UAM-1O(cp) sample (red), the UAM-1(op) sample soaked in DCM and vacuum-dried at room temperature overnight (blue trace). The latter sample is also shown after being gently touched with a needle (green). For details on the mechanical perturbation, please refer to the accompanying video. 15
Figure S14 Local energy profile as a function of volume for closed phase (left) and open phase (right). 16
Figure S15 Schematic representation of two considered transition mechanism. The initial structure cp1 is presented on the left with two oxygens labeled as *a* and *b*, in the middle transition path (top) with breaking the Zn–O(*b*) bond and rotation of COO group around Zn–O(*b*) bond, and transition path (bottom) with breaking both Zn–O bond and formation of new Zn–O(*a*) bond, on the right are resulting structures with different resulting non-bonded oxygens 17
Figure S16 Representation of linkers extracted from periodic UAM-1 structures: a) UAM-1O-cp without bond rearrangement, b) UAM-1O-cp with rearranged bonds, c) UAM-1O-meta, d) UAM-1O-op, e) UAM-1S-cp, and f) UAM-1S-op. Angles defined in the analysis: "angle1"=∠ada, "angle2"=∠bdb, and "angle3"=∠cdc. 18
Figure S17 Graphical representation illustrating the definition of the out-of-plane angle used in the analysis (yellow angle). The brown arrows indicate distance which was varied to examine planarity of the aromatic ring. Hydrogen atoms are hidden for clarity. 18
Figure S18 Visualization of HOMO orbitals for oba (a, c) and sba (b, d) linkers. Bottom images (c, d) represent alternative orientations for clarity. 19
Figure S19 Visualization of LUMO orbitals for oba (a, c) and sba (b, d) linkers. Bottom images (c, d) represent alternative orientations for clarity. 19
Figure S20 Capillary with loaded UAM-1O, after time-resolved PXRD measurement. Black dots indicate destruction of part of the sample. 19
Figure S21 Fractions of the open pore phase for UAM-1O based on in situ synchrotron PXRD measurement normalized peaks intensity and the trend line was obtained by fitting the experimental data to the KJMA equation a) Full data; b) modified data by cutting ; c) Color maps of in situ PXRD patterns collected upon CO_2 adsorption on UAM-1O 20
Figure S22 Fractions of the open pore phase for UAM-1S in different pressures and color maps of in situ PXRD patterns collected upon CO_2 adsorption (195 K). 21
Figure S23 a) Time evolution of α at 195 K and 0.2, 0.4 and 0.8 kPa·s^{-1}. **b**) Pressure dependence of α in **a**. 21
Figure S24. a) Time evolution of α at 195 K and 0.2, 0.4 and 0.8 kPa·s^{-1}. **b)** Pressure dependence of α in a. 22
Figure S25 Attempt at deriving the structural transition rate model for UAM-1O according to Watanabe's work[12]. a) Pressure dependence of the structural transition rate at various α values. **a, b**) Example of analysis for α = 0.5, where the derivative values of α with respect to pressure are extracted from time-resolved in situ X-ray powder diffraction (TRXRD) results at 0.005, 0.2, 0.4, and 0.8 kPa s^{-1}. The dα/dP values are obtained by multiplying the pressurization rate. The solid line in panel b is a linear least-squares fit, where the slope represents the function g(α) and the x-intercept represents h(α). **c**) Relationship between the function g(α) obtained for different α values. **d**) Relationship between α and the function h(α) obtained for different α. 22
Figure S26 Diffraction images from time-resolved studies 23

Table 1 Summary of the microED data collection, reduction, and refinement statistics of the compounds. 24
Table 2 The crystallographic data of metastable phase UAM-1O op. 25
Table 3 Volume of equilibrium structures (Vin), their energy ($EVmin$), EOS bulk modulus ($B0$), and the derivative of bulk modulus with respect to pressure ($B0'$) 25

Experimental and Theoretical Procedures

X-ray Crystallography: The data have been deposited in the Cambridge Crystallographic Data Collection (CCDC) with deposition numbers CCDC 2419835, 2247315 and 2247321. These data can be obtained free of charge via www.ccdc.cam.ac.uk/data_request/cif, by emailing data_request@ccdc.cam.ac.uk, or by contacting The Cambridge Crystallographic Data Centre, 12 Union Road, Cambridge CB2.

Obtaining a metastable UAM-1O(op) phase: After the synthesis of UAM-1O and UAM-1S, DMF was decanted from the crystals and DCM was added. After 3 hours, DMC was decanted and fresh DCM was added and the materials were left in DCM overnight. The next day, DCM was decanted and replaced with fresh DCM. Next, repeat this procedure for another 3 days. The crystal suspension in DCM was transferred using a Pasteur pipette onto a watch glass. The watch glass was left exposed to air at room temperature. After a few minutes, when the DCM had completely evaporated, it was noticed that some of the crystals remained intact. It is worth noting that the crystals that are considered to be an empty open phase are very sensitive to touch, which means that even a slight touch with a needle causes them to fragment. Despite this behavior, it was possible to perform an SC-XRD measurement.

Scanning electron microscopy: High-magnification imaging was performed using a ZEISS AURIGA 60 FE-SEM (field emission scanning electron microscope). The samples were coated with a 10 nm gold layer to improve charge dissipation. Imaging was conducted in secondary electron (SE) mode with accelerating voltages ranging from 2 to 20 kV.

Powder x-ray diffraction sample preparation and data collection

1. After synthesis, the crystals were washed with hot DMF to remove ligands. The sample cooled to room temperature and DMF was replaced with DCM. After the sample had cooled to room temperature, DMF was replaced with DCM. In the next steps, DCM was poured off and replaced with fresh one, this procedure was repeated 3 times a day for two days. During the solvent exchange, a color change from yellow to pale yellow/colorless was observed.

2. For the measurement of UAM-1O(op)@DCM, a Bruker D8 Quest (Cu radiation) diffractometer was used, due to the high volatility of DCM, the measurement was performed in a capillary. (**Figure S8c**) In order to capture the UAM-1O(op)M phase, the crystals in DCM were transferred to a background less measuring holder and the solvent was allowed to evaporate. After this time, the powder diffraction pattern was measured (BRUKER AXS D8 Advance. After the measurement (the measurement lasted approx. 40 min), another measurement of this sample was performed to check for possible structural changes over time. In the next step, the sample was gently touched with a needle. After that, the PXRD was measured again (**Figure S8d**).

MicroED sample preparation and data collection:

1. After the synthesis of UAM-1O and UAM-1S, DMF was decanted and DCM was added. After 3 hours, DMC was decanted and fresh DCM was added and the materials were left in DCM overnight. The next day, solvent was replaced with fresh DCM. After 3 hours, DCM was decanted and the crystals were transferred to a Schlenk apparatus. The systems were heated at 80ºC for 3 hours under vacuum. Next DCM was added, then DCM was decanted and the sample was activated at 80ºC under vacuum for about 3 hours. The procedure was repeated two times more, thus obtaining fine-crystalline UAM-1O material suitable for MicroED measurement. The sample size was monitored by SEM and TEM imaging (**Figure S6**).

2. However, in the case of UAM-1S, the obtained crystals did not produce a good diffraction pattern. Considering that the rapid solvation and desolvation processes may have caused crystal damage of UAM-1S, we conducted two cycles of CO_2 adsorption/desorption at 195 K using a Bell Sorp MAX device. It allow us to produce some of 200nm non defective crystals suitable for MicroED. The sample size was monitored by SEM and TEM imaging (**Figure S6**).

3. Grids for MicroED data collection were prepared by directly applying a pinch of powdered crystals to a freshly glow discharged lacy carbon 200 mesh Cu grid. Following that, the grids were clipped at room temperature (RT) and transferred to the microscope for data collection. Grids were then cooled while the microscope was cooling under the vacuum. A Thermo Fisher Scientific Glacios cryo transmission electron microscope (TEM) equipped with a field emission gun operated at 200 kV and a stage holder temperature of 81 K was used for data collection on one single crystal **(Figure S1)** of each compound. The microscope was equipped with a Thermo Fisher Scientific CETA-D detector, an autoloader with twelve grid holders and EPU-D software for automated data collection. A 50 μm condenser aperture, spot size 11, and gun lens 8 were set and diffraction datasets were collected

under parallel illumination condition with very low dose (3.6 eÅ^{-2}). The crystal was continuously rotated from -60° to +60° under the paralleled beam. The microscope was set in a diffraction mode and the camera collecting continuously in a rolling shutter mode with hardware binning 2 and exposure time 0.5 s. The collected images were saved in SMV format built in the EPU-D software. The crystallographic data are summarized in the table. (**Table S1**).

Data Processing & refinement details: Frames were indexed and integrated in XDS[1] and the intensities were converted to SHELX format using XPREP. The structures were solved in SHELXT[2]. All the structural refinements were performed in Olex2[3] using olex2.refine in the kinematical diffraction theory approach. In the refinement the following weighting scheme was applied: w=1/[σ2(Fo2)+(0.2P)2], where P=(Fo^2+2Fc^2)/3. The structure was deposited in the Cambridge Crystallographic Data Centre (CCDC) under the accession number 2408974 and 2408973.

Single crystal X-ray diffraction. Reflection intensities for metastable phase were collected on a Rigaku SuperNova diffractometer equipped with a Cu microfocus source (λ=1.54178 Å) and a 135 mm Atlas CCD detector, at 135K. The sample temperature were controlled with an Oxford Instruments Cryojet Controller. Data collection, data reduction, and analytical numeric absorption collection using a multifaceted crystal model were performed with the CrysAlis^{Pro} software.[4,5] The crystal structure was solved by direct methods using SHELXT[6] and refined using a full matrix least-squares procedure based on F^2 with SHELXL[7], within the graphical user interface X-Seed.[8] Non-hydrogen atoms were refined anisotropically. Hydrogen atoms were placed at calculated positions and refined using riding models, and their isotropic displacement parameters were assigned values 20% higher than the isotropic equivalent for the atoms to which they are attached.

To assign the electron density in the voids following crystal activation and to account for residual solvent molecules, a solvent mask routine using OLEX2[9,10] was employed. Analysis determined the number of electron count to be 64 electron per unit cell (8 ē per asymmetric unit cell and 17 ē per formula unit). Since the crystals were obtained from DCM, which contributes 42 ē, this suggests that the estimated electron count of 64 ē in an accessible void volume of 4912 Å^3 corresponds to 1.5 DCM molecules per unit cell (64/42~1.5). The same procedure was then applied to the solvated open phase, revealing a density of 1030 electrons per unit cell (258 ē per asymmetric unit and 515 ē per formula unit).

Time-resolved in situ PXRD experiments were conducted at P23 in situ diffraction and imaging beamline of PETRA III synchrotron (DESY). Monochromatic irradiation with E = 20.0 keV (λ = 0.619921 Å) was used in all experiments. Reflection intensities were measured using a PILATUS 1M (DECTRIS) detector. The distance between the sample and detector is 45 cm. In a typical experiment, PXRD patterns were collected with 10 Hz rate during the 300 – 3600 s, integration of the 2D diffraction images was done by the pyFA[11] software. The experimental setup consists of volumetric adsorption instrument BELSORP-max (Microtrac MRB), used as a gas handling system, was connected to the customized home-built in situ glass capillary adsorption cell. The dosing of CO_2 to the cell and PXRD synchrotron measurements were synchronized by a TTL-trigger. The temperature of the experimental cell was controlled at 195K by Oxford Cryostream 700 Series Cryostream Cooler . In the typical experiment, the desired pressure of carbon dioxide was built in the standard volume part while the cell was completely evacuated to the vacuum degree of $p \leq 1$ Pa.
All raw data needed to reproduce the data analysis are available at:
https://researchportal.amu.edu.pl/info/researchdata/UAM2e8a966edb0f495c94ff9e1a3db0f4af/.

Time-resolved in situ PXRD experiments during the controlled pressure increase on UAM-1O and UAM-1S were conducted at P23 in situ diffraction and imaging beamline of PETRA III synchrotron (DESY). Monochromatic irradiation with E = 20.0 keV (λ=0.619921 Å) was used in all experiments. Reflection intensities were measured using PILATUS 1M (DECTRIS) detector, installed 45 cm back from the sample. In the typical experiment PXRD patterns were collected with 2 Hz rate.

The experimental setup consists of specialized gas dosing system, connected to the customized home-built capillary holder. The gas dosing system was connected with in situ cell using 1/8 inch stainless tube. The nitrogen cryojet was used to cool down the measuring range of the capillary to 195 K. Initially the samples cell was first exposed to dynamic vacuum at 298 K for at least 5 minutes and after the cryo nozzle was moved 7 mm to the capillary. After that the CO2 gas was introduced with the constant pressure ramps of 0.2 kPa/s, 0.4 kPa/s and 0.8 kPa/s. The data were used for the estimation of the switching transition using the procedure, introduced by Watanabe et al.[12]

Initial Structures and Optimization Procedures: The initial open phase structures of both UAM-1 variants for open phase were constructed based on experimentally recorded structures. In the next step, the cp1 structures were initially obtained from molecular modeling simulations under 1.5 GPa pressure using the Universal Force Field[13] for the description of interactions. Subsequently, all structures (both for both UAM-1O and S, and for op and cp_1) were fully optimized using the plane-wave density functional theory (DFT) method implemented in the Vienna Ab Initio Simulation Package (VASP version 6.4.0).[14–16] We used the Perdew-Burke-Ernzerhof (PBE) exchange-correlation density functional[17] together with Grimme's D3 dispersion correction with Becke-Johnson damping.[18,19] For full optimization (atom positions, cell vectors and cell volume) we used VASP setting ISIF = 3. A plane-wave basis set with an energy cutoff of 700 eV was used and the structure was optimized until the electronic energies and forces on the atomsc onverged within 10−6 eV and 0.02 eV/Å respectively. Only Γ-point was used to sample the Brillouin zone, due to the large unit cell of the structure. In the next step, to avoid any effects resulting from Pulay stress, the optimized structures were scaled into slightly larger and smaller volumes, and were then optimized again as before, but with the volume kept fixed (VASP setting ISIF=4). Subsequently, the energy of configuration at given volume was obtained from single-point calculations. By fitting the Rose-Vinet equation of state[20,21] (Figure S11) we were able to calculate the volume (V_{min}) and energy ($E_{V_{min}}$) of the structures at the local energy minimum, along with their bulk modulus (B_0) and its derivative with respect to pressure (B_0') (Table S3). Subsequently, structures at varying volumes were generated based on the structures at the local minimum energy volume using an in-house Python script. This involved interpolation and extrapolation of cell vectors and atomic positions between the op and cp1 variants.

Nudged Elastic Band Calculations: To investigate the processes of bond formation and breaking during UAM-1O transition, we employed the solid state nudged elastic band (ssNEB) methodology[22] as implemented in Transition State Library for ASE (TSASE).[23] The initial configuration was derived from the optimized cp1 and cp2 structures, which were created through manual re-bonding of the cp1 structure and subsequent full optimization. Seven images were utilized, and minimization was conducted until the force converged to 0.01 eV/Å. The cluster model of the Zn-node and surrounding linkers (comprising four oba^{2-} linkers and two TzTz linkers) was extracted from the periodic UAM-1O structure, oba^{2-} linkers were truncated with Li^+ ions to maintain overall charge neutrality and fully optimized. To maintain overall charge neutrality, the oba^{2-} linkers were truncated with Li^+ ions and fully optimized. The Li^+ ion was selected for use in this instance due to the fact that the Li atom is located in a symmetrical position at the centre of the linker, providing an optimal anchor point while simultaneously allowing for the rotation of the entire group in the subsequent step of the calculation. In the subsequent step, the two oxygens were re-bonded in accordance with the shift variant illustrated in Figure 2 of the main text. All atomic positions were optimized with the fixed positions of the Li^+ ions and the terminal N atoms of the TzTz linkers. This approach was employed to avoid cluster collapse, while simultaneously allowing for more degrees of freedom than those imposed by the confinement of the crystal lattice. Following the optimization of the cp_2 and cp_1 cluster model, NEB calculations were performed using ORCA 5.0.4 software[24] with the BP86 functional, the def2-TZVP basis set for Zn atoms, and the def2-SVP basis set for the remaining atoms. As previously, the D3(BJ) dispersion correction was employed in conjunction with the RI-J approximation. In the cluster approach, 17 images were utilized, and the default value for force convergence was employed, namely 1.00e-03 Eh/Bohr. All simulation input files needed to reproduce calculations are available on Zenodo. Mazur, B., Kuchta, B., & Formalik, F. (2025). Computational Supplementary Data for Paper "How a Single Atom Influences the Spatiotemporal Response of Flexible MOFs: Insights from Theory and Experiment" [Data set]. Zenodo. https://doi.org/10.5281/zenodo.14749562.

Linkers Analysis: The structural analyses presented in this work began with experimentally determined closed phase (cp), open phase (op), and metastable (meta) forms of UAM-1O, and cp and op forms of UAM-1S. Additionally, theoretically-derived structure of UAM-1O cp variant, without rearranged Zn–O bonds, was investigated. Initial linker configurations (oba^{2-} and sba^{2-}) were extracted directly from these periodic structures and capped with Li^+ ions to maintain charge neutrality. Geometry optimizations of these linker fragments were performed using density functional theory (DFT) at the r^2SCAN-3c/def2-mTZVPP level of theory.[25] Following initial geometry optimization, the flexibility of both oba and sba linkers was systematically assessed. Linker flexibility analyses involved geometric scans of the out-of-plane angle at the bridging atom (O or S) in the range of 175°–190°. The out-of-plane angle was defined between the aromatic ring plane and the vector connecting the bridging atom (O or S) to its adjacent carbon atom (**Figure S16 and Figure S17**). Throughout these scans, positions of carbon atoms defining this angle were constrained, while all remaining atomic coordinates were allowed to optimize freely. Additionally, linker deformation under structural compression was explored by varying the distance between the aromatic ring carbon atoms and the corresponding carboxyl group carbon atoms within 4.000–4.267 Å (oba^{2-}) and 4.000–4.282 Å (sba^{2-}) (**Figure S16 and Figure S17**). Subsequently, carbon atom positions were constrained, and remaining atomic coordinates optimized, to isolate aromatic ring deformation upon compression. Quantitative assessment of linker planarity employed a least-squares plane-fitting procedure based

on singular value decomposition (SVD). First, the centroid of carbon atoms in aromatic and carboxyl groups was calculated, and atomic coordinates were centered relative to this centroid. SVD then yielded the best-fit plane, from which deviations of individual atoms were calculated and expressed as root-mean-square (RMS) deviations to quantify planarity. Electronic properties of optimized linkers were analyzed through HOMO/LUMO orbital calculations (using ORCA LARGEPRINT option) and visualized using IboView software.[26,27]

The overall planarity was quantified by computing the root-mean-square (RMS) of these distances:

$$RMS = \sqrt{\frac{1}{N}\sum_{i=1}^{N}\left((\boldsymbol{r_i} - \boldsymbol{r_{centroid}}) \cdot \boldsymbol{n}\right)^2}$$

Figures

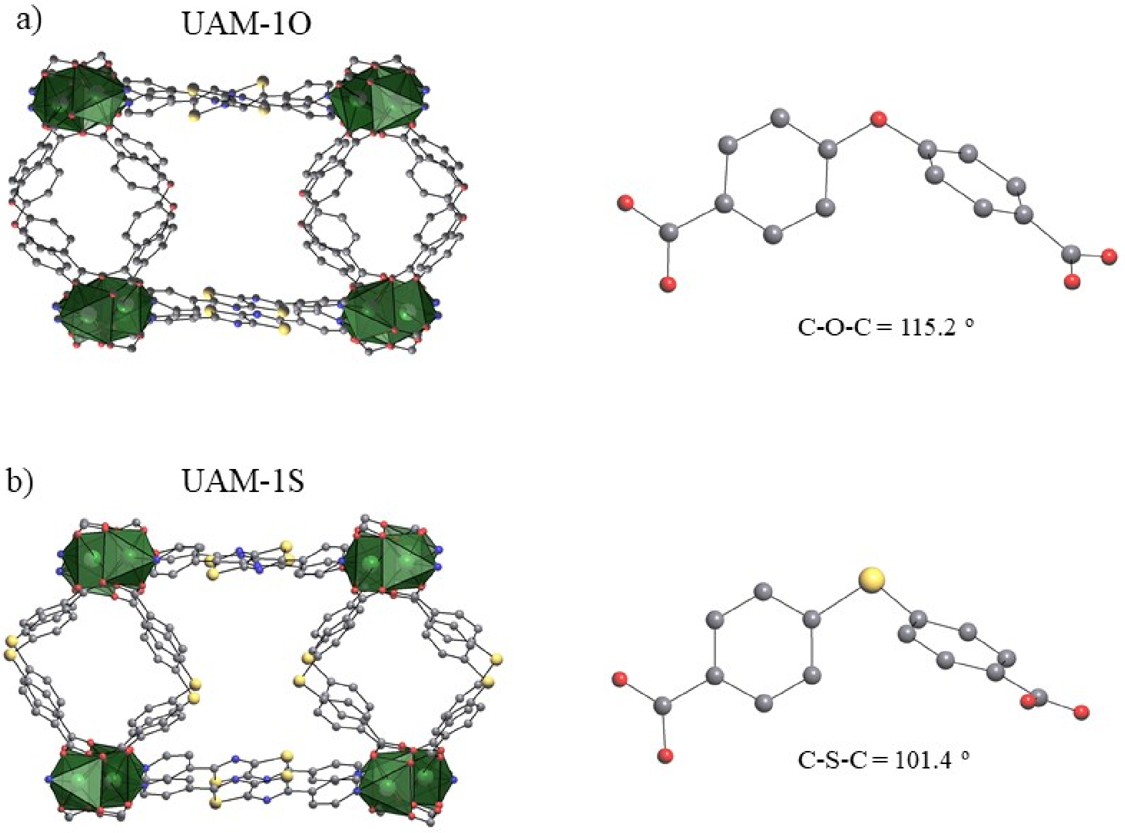

Figure S1 Pore geometry (a and b) and carboxylate ligand along with the C-X-C angle (X:O or S): Top row UAM-1O(op); bottom row UAM-1S(op).

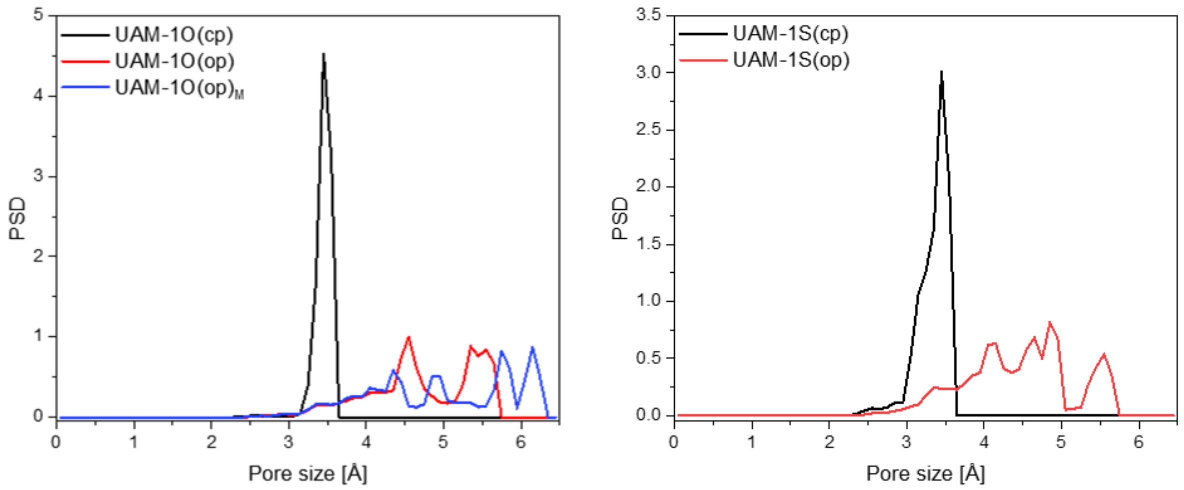

Figure S2 Pore size distribution of different phases of UAM-1O (left) and UAM-1S (right).

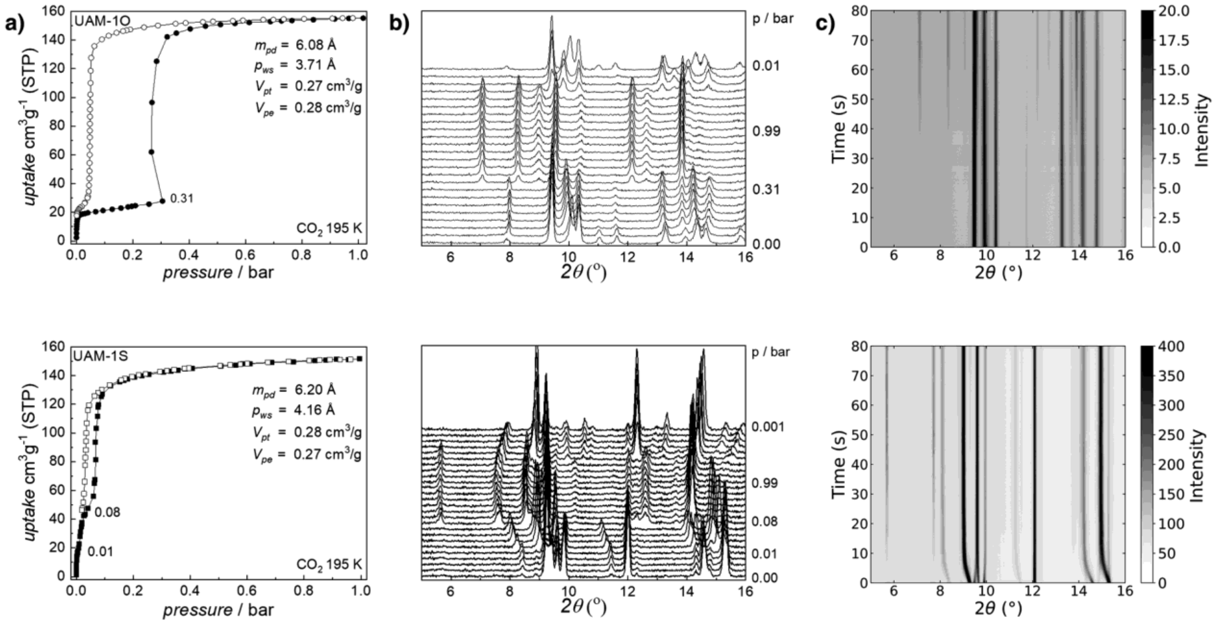

Figure S3 Mechanistic understanding of CO_2-driven structural transformation in UAM-1X (O – top row; S – bottom row): (a) CO_2 adsorption (full symbols) and desorption (open symbols) at 195 K juxtaposed with (b) corresponding *in situ* PXRD patterns collected at selected pressure (λ = 1.540599 Å); (c) *in situ* time-resolved PXRD during the CO_2 adsorption at 195 K. m_{pd}: maximum pore diameter; p_{ws}: pore window size; V_{pt}, theoretical pore volume; and V_{pe}, experimental pore volume for CO_2 adsorption calculated at ~0.99 bar according to the Gurvich's rule. Reproduced with permission from the Royal Society of Chemistry.[28]

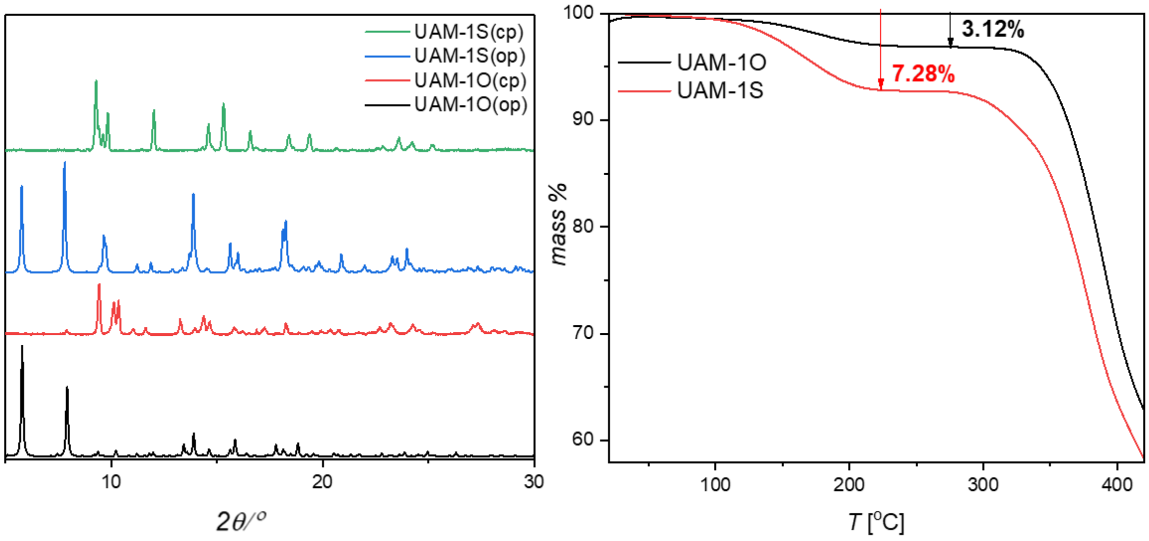

Figure S4 Powder diffractograms of open and closed phases of UAM-1X. (left) Thermal gravimetric analysis UAM-1O (red) UAM-1S (black), the chart shows the percentage of weight loss (right).

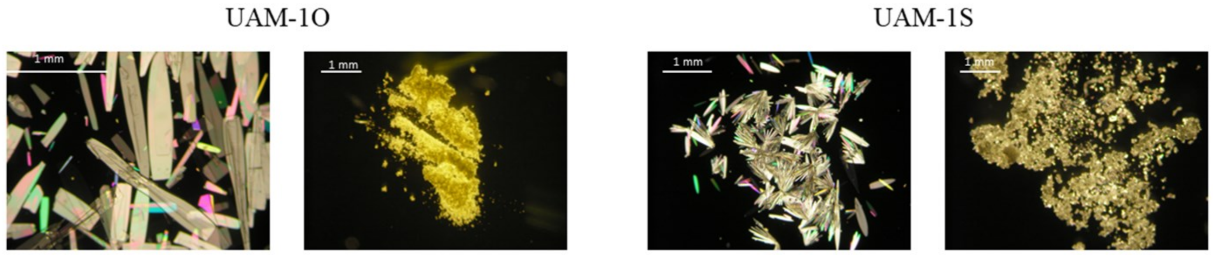

Figure S5 UAM-1X (X: O or S) microscopic images of crystals (with scale bar) after synthesis (left) and after activation (right; DCM exchange; 80 °C and vaccum) taken under polarized light.

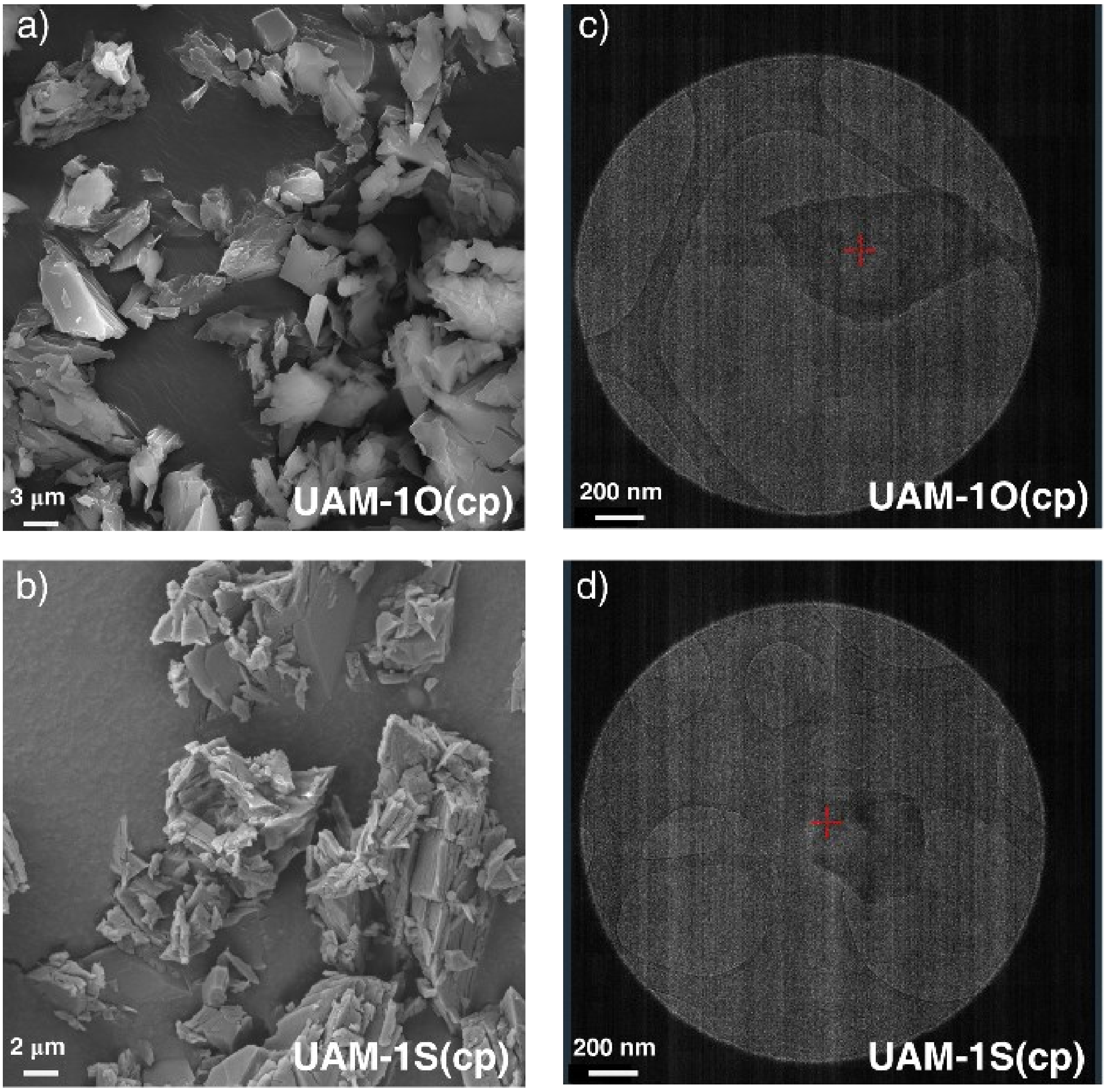

Figure S6. (**a**) and (**b**) Scanning electron microscope images of UAM-1S(cp) and UAM-1O(cp), along with corresponding (**c**) and (**d**) TEM images of microcrystals used for microED. UAM-1O(cp) was prepared through five desolvation-solvation cycles of UAM-1(op) in DCM, while UAM-1S(cp) was obtained through adsorption-desorption cycles of CO2 at 195 K. UAM-1O(cp) = $Zn_2(oba)_2TzTz$, and UAM-1S(cp) = $Zn_2(sba)_2TzTz$.

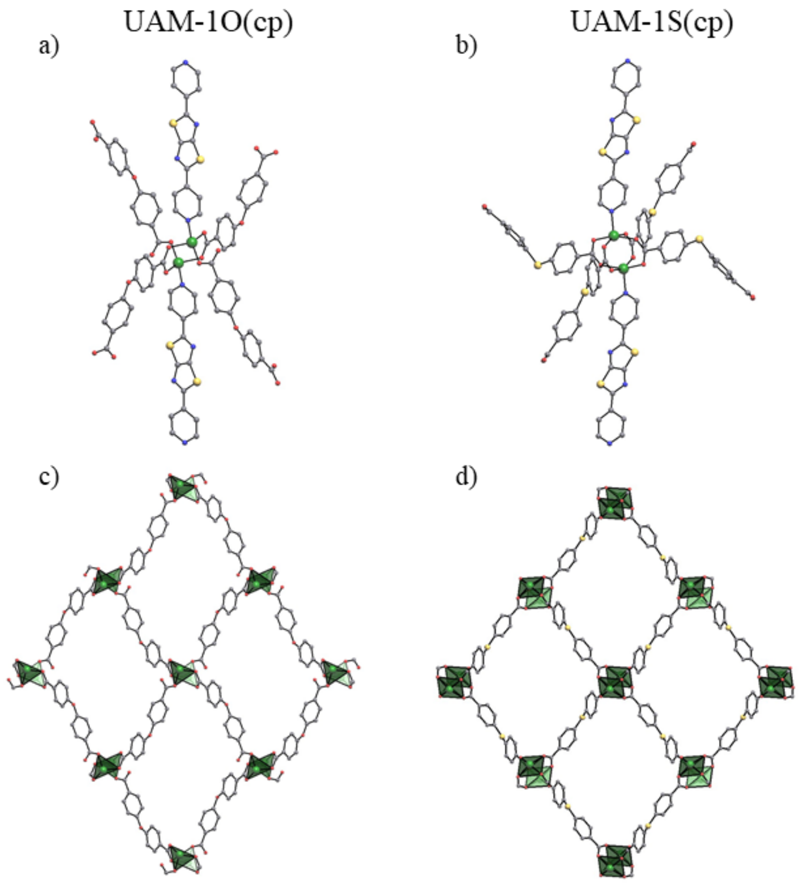

Figure S7 a) UAM-1O(cp) coordination environment; b) UAM-1S(cp) coordination environment; c) 2D layer formed by $Zn(oba)_2$ and d) $Zn(sba)_2$ (projected along the [100] direction).

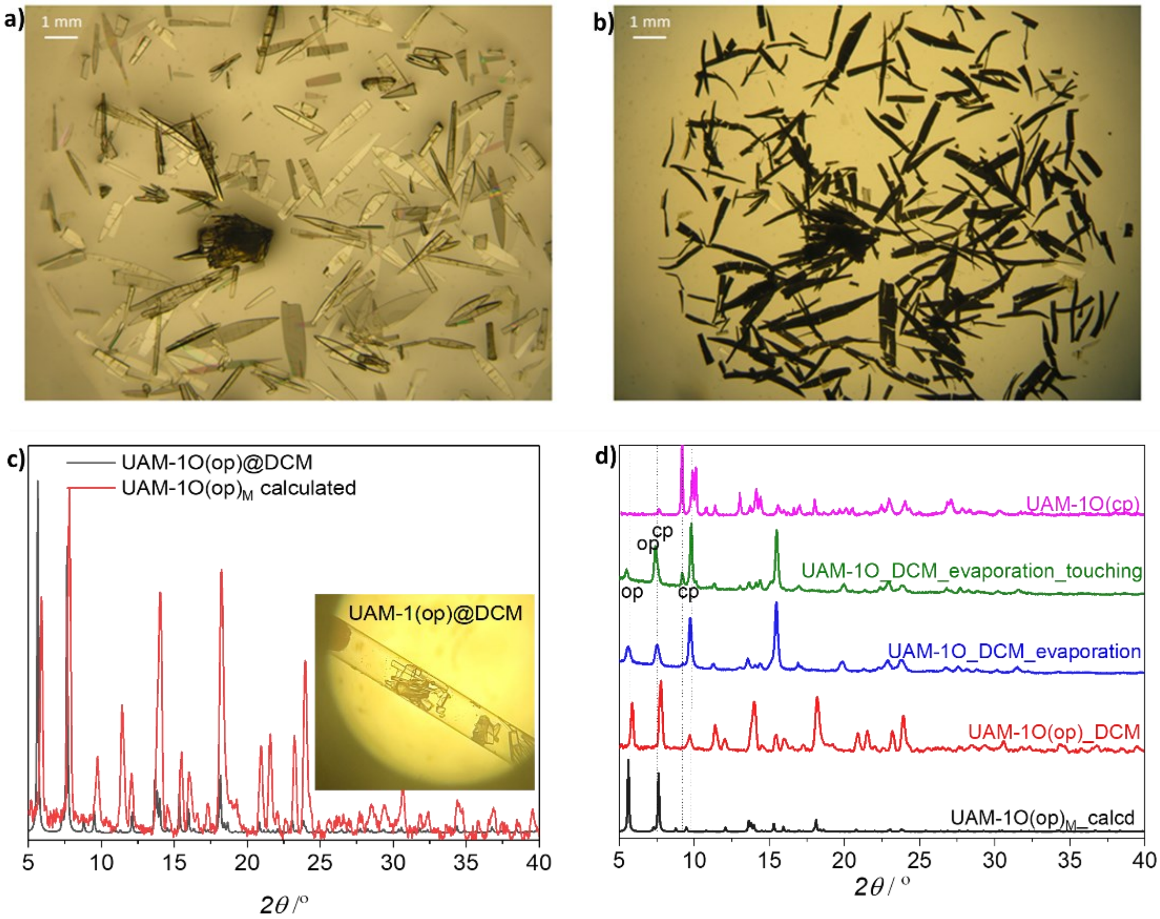

Figure S8 Microscope photos (with scale bar) of **a**) UAM-1O soaked in DCM (left) and the same crystals after evaporation of **b**) DCM in ambient conditions (right); most of the crystals have been defragmented and powdered, but there are some crystals visible that are unchanged and are likely to be UAM-1O(op)$_M$. **c**) Comparison of PXRD patterns of UAM-1O after soaking in DCM with the calculated patterns of UAM-1O(op)$_M$; due to fast DCM evaporation rate, the measurement was done in the closed capillary. **d**) Evolution of PXRD patterns of UAM-1O through the following steps: *1)* soaking in DCM (red), *2)* evaporation at ambient conditions (blue), and *3)* touching (green), compared with the calculated patterns of UAM-1O(op)$_M$ (black) and the experimental pattern of UAM-1(cp), magenta. For details on the mechanical perturbation, please refer to the accompanying video.

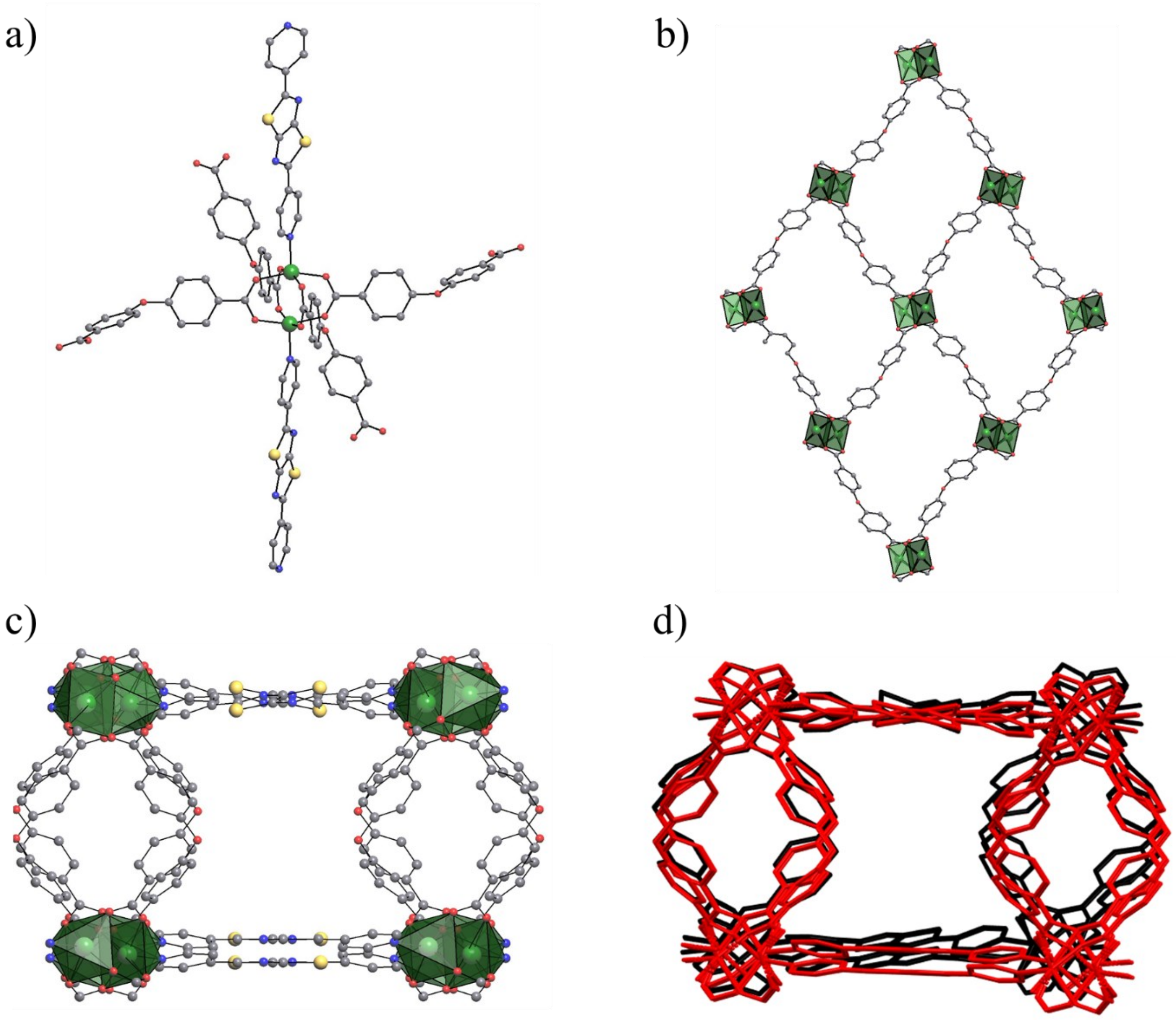

Figure S9 UAM-1O(op)$_M$: a)coordination environment, b) layer composed of Zn(oba)$_2$ and d) the three-dimensional UAM-1O(op)M framework. d) Overlay of UAM-1O(op), black, with UAM-1(op)$_M$, red, structures along the z axis.

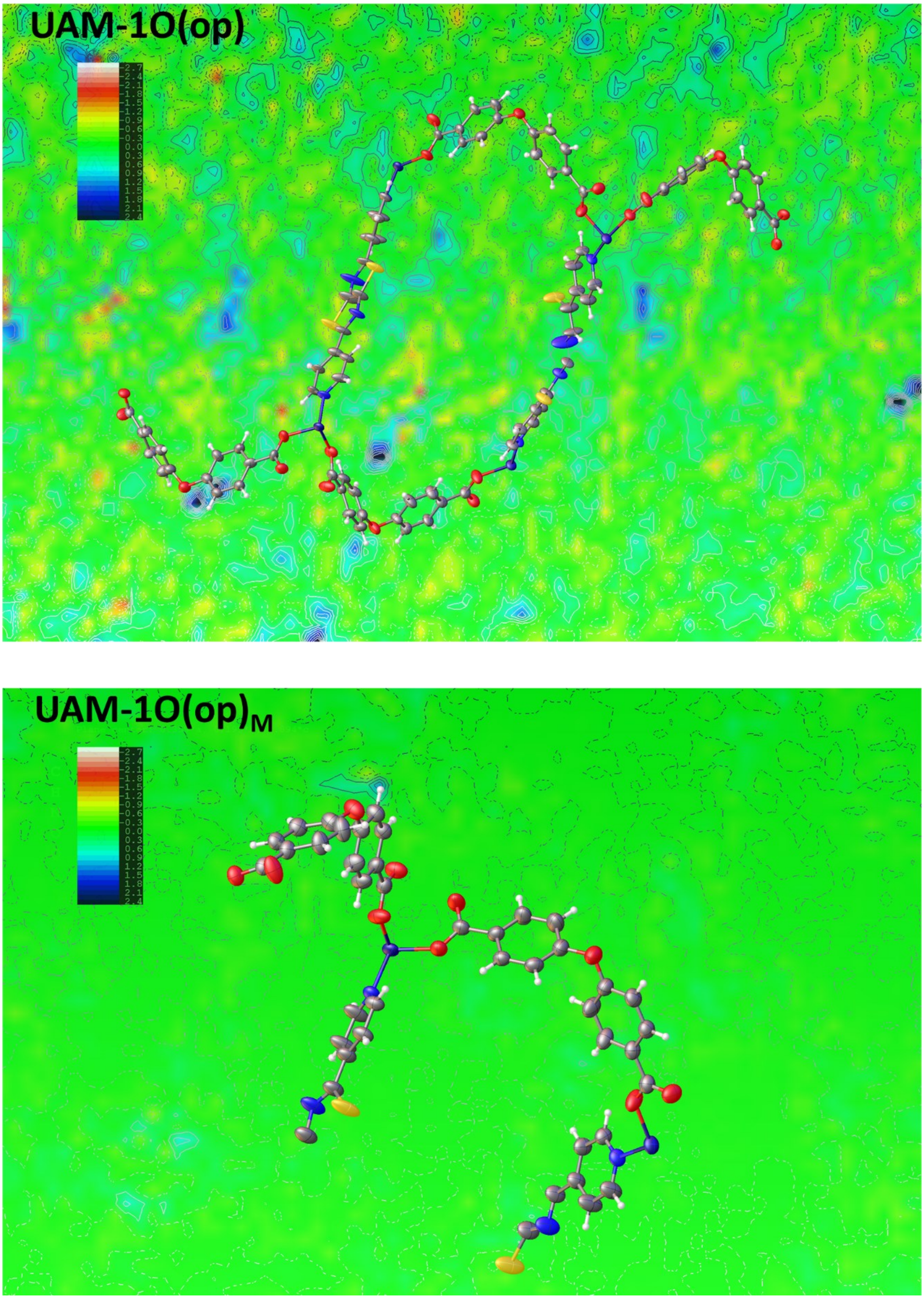

Figure S10 Electron density maps for UAM-1O(op) and for UAM-1O(op)$_M$.

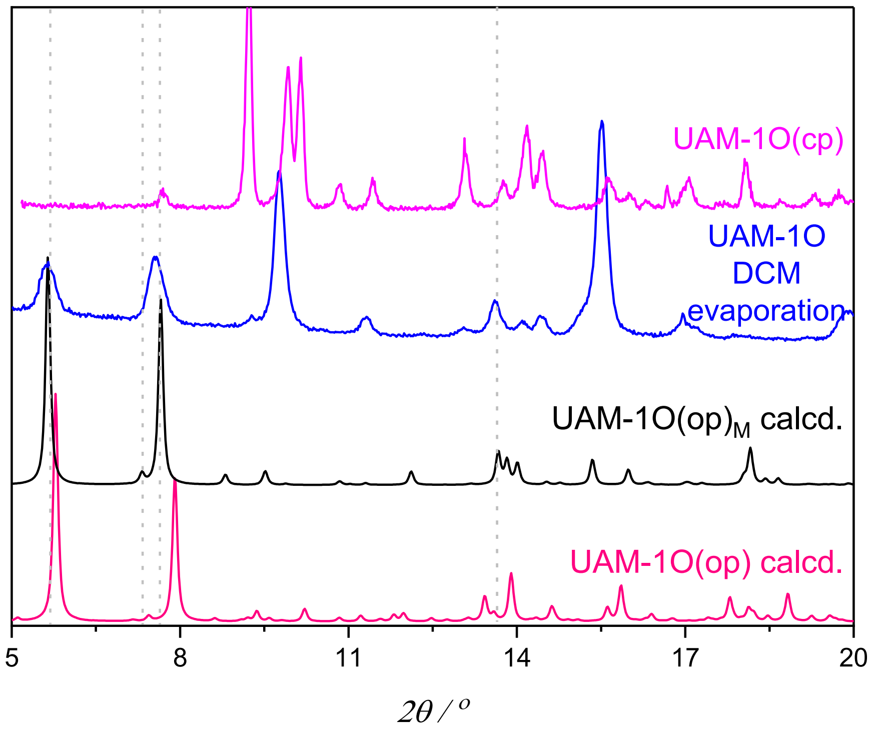

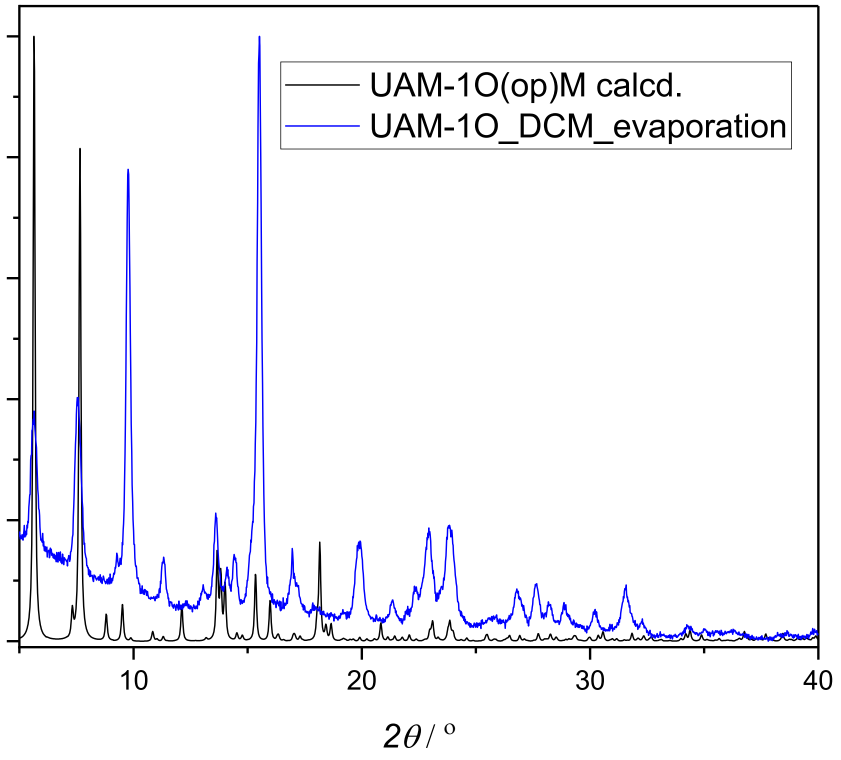

Figure S11 The comparison of calculated PXRD of UAM-1(op), UAM-1(op)M and UAM-1(cp) with the bulk sample after the DCM evaporation at ambient conditions (top). The comparison of calculated UAM-1(op)M with the bulk sample after the DCM evaporation (down).

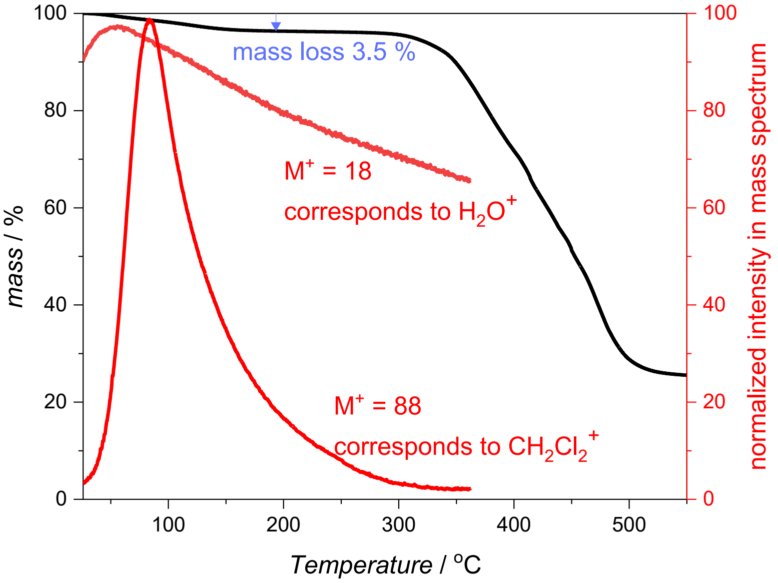

Figure S12 TG-MS analysis of the bulk sample after the DCM evaporation.

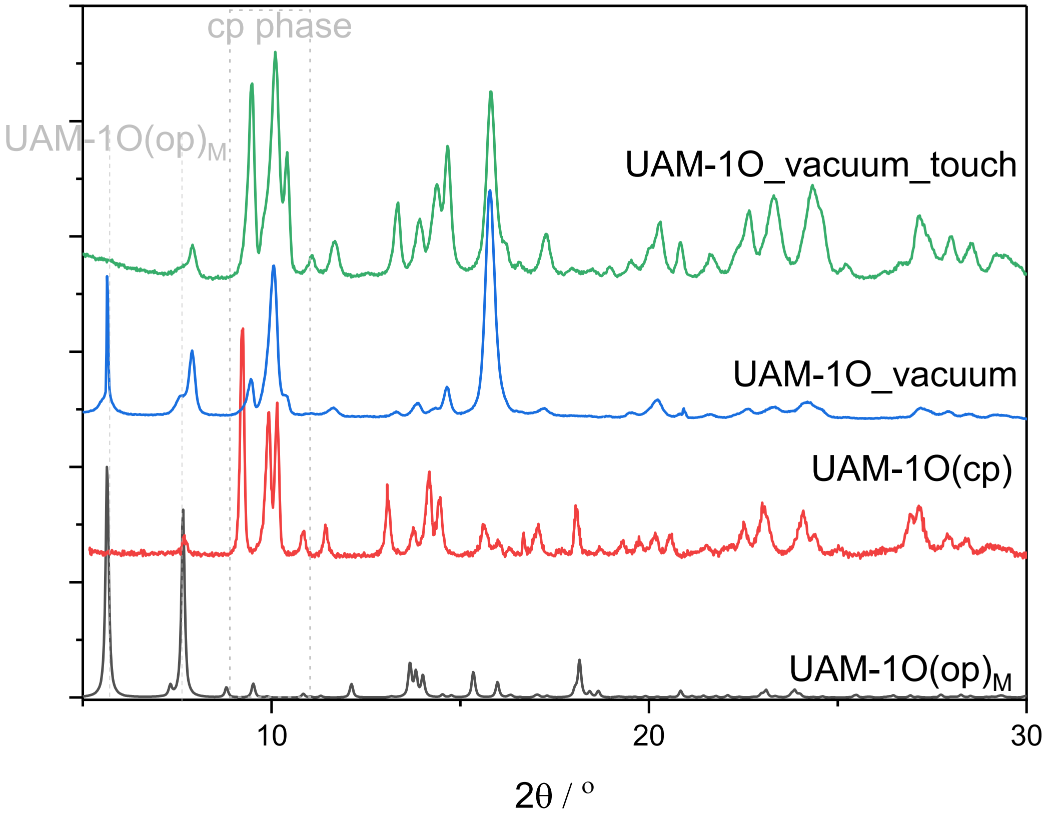

Figure S13 Comparison of the PXRD patterns of the calculated UAM-1O(op)$_M$ with UAM-1O(cp) sample (red), the UAM-1(op) sample soaked in DCM and vacuum-dried at room temperature overnight (blue trace). The latter sample is also shown after being gently touched with a needle (green). For details on the mechanical perturbation, please refer to the accompanying video.

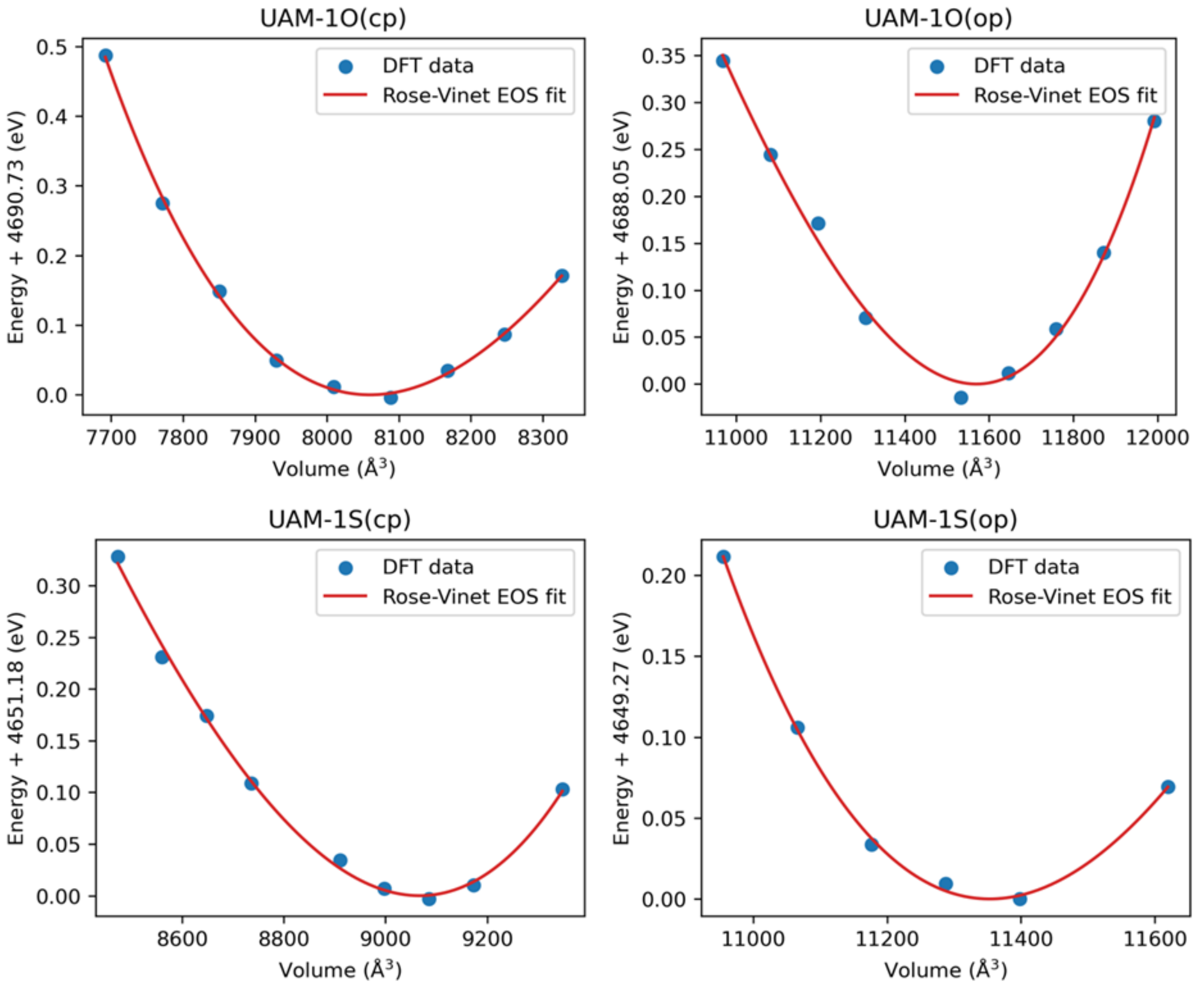

Figure S14 Local energy profile as a function of volume for closed phase (left) and open phase (right).

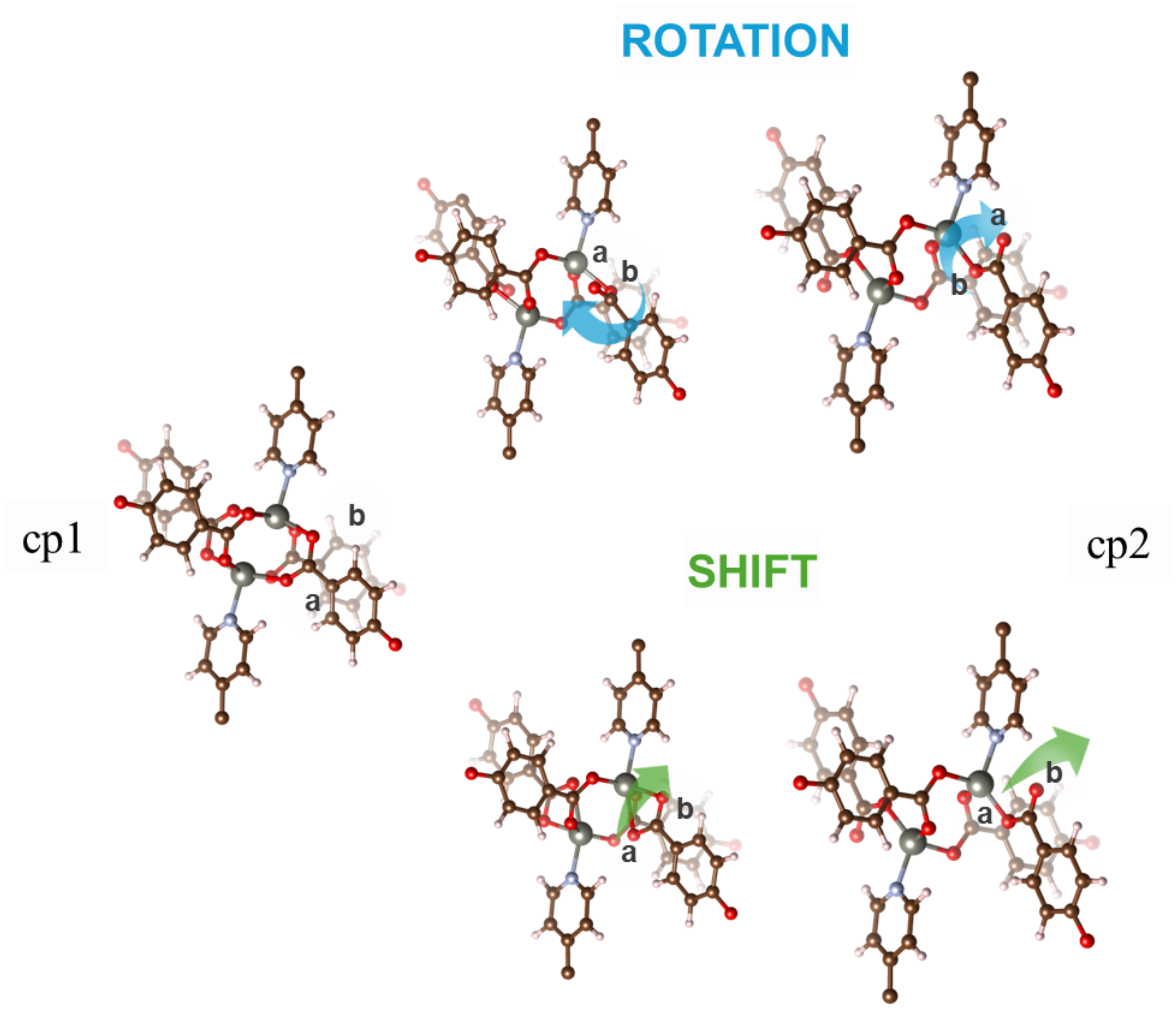

Figure S15 Schematic representation of two considered transition mechanism. The initial structure cp1 is presented on the left with two oxygens labeled as *a* and *b*, in the middle transition path (top) with breaking the Zn–O(*b*) bond and rotation of COO group around Zn–O(*b*) bond, and transition path (bottom) with breaking both Zn–O bond and formation of new Zn–O(*a*) bond, on the right are resulting structures with different resulting non-bonded oxygens.

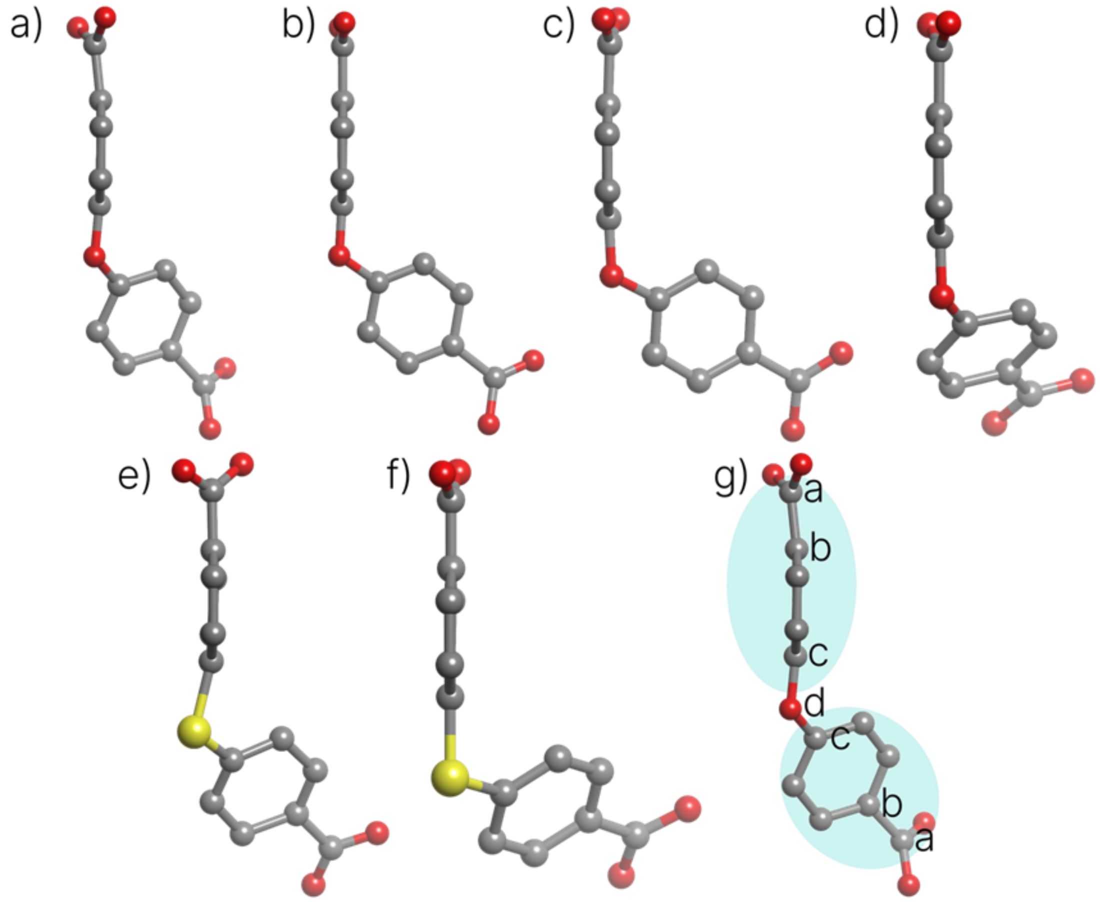

Figure S16 Representation of linkers extracted from periodic UAM-1 structures: a) UAM-1O-cp without bond rearrangement, b) UAM-1O-cp with rearranged bonds, c) UAM-1O-meta, d) UAM-1O-op, e) UAM-1S-cp, and f) UAM-1S-op. Angles defined in the analysis: "angle1"=∠ada, "angle2"=∠bdb, and "angle3"=∠cdc.

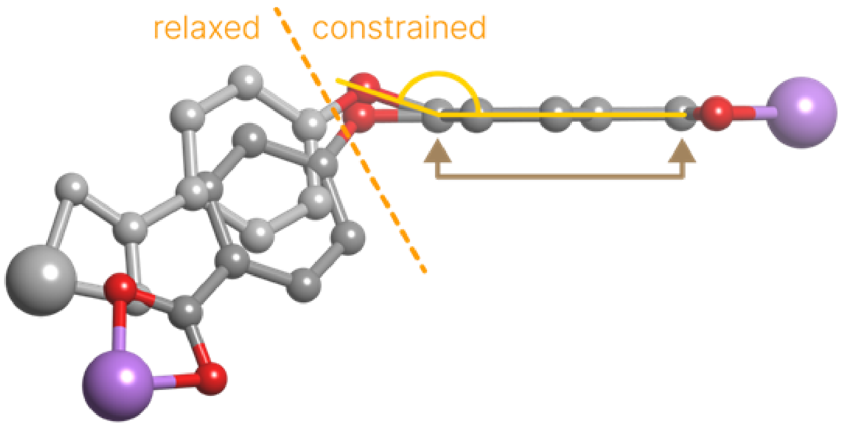

Figure S17 Graphical representation illustrating the definition of the out-of-plane angle used in the analysis (yellow angle). The brown arrows indicate distance which was varied to examine planarity of the aromatic ring. Hydrogen atoms are hidden for clarity.

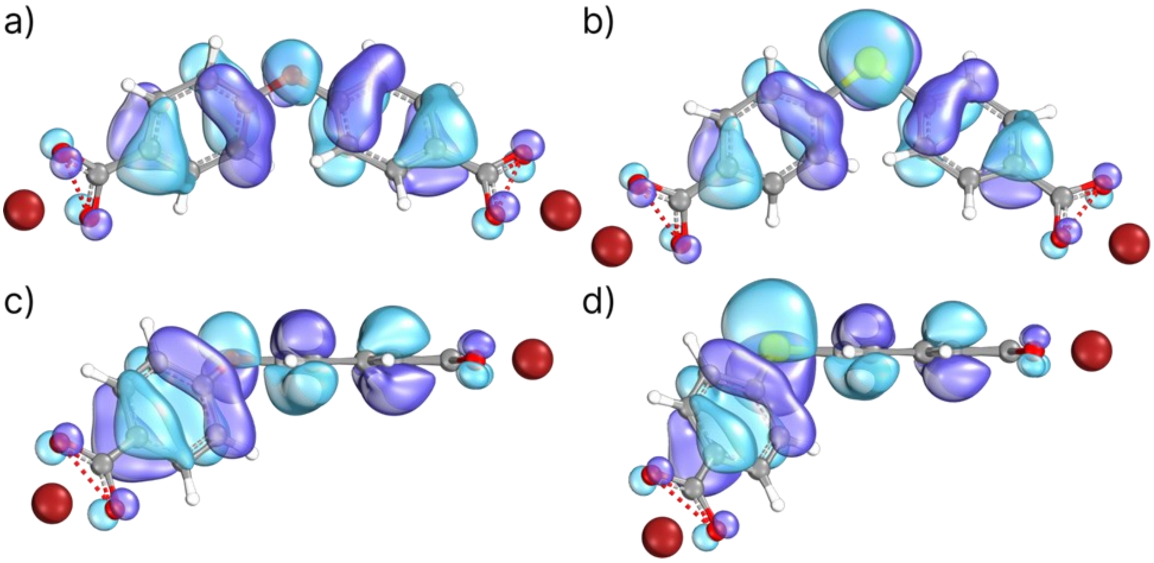

Figure S18 Visualization of HOMO orbitals for oba (a, c) and sba (b, d) linkers. Bottom images (c, d) represent alternative orientations for clarity.

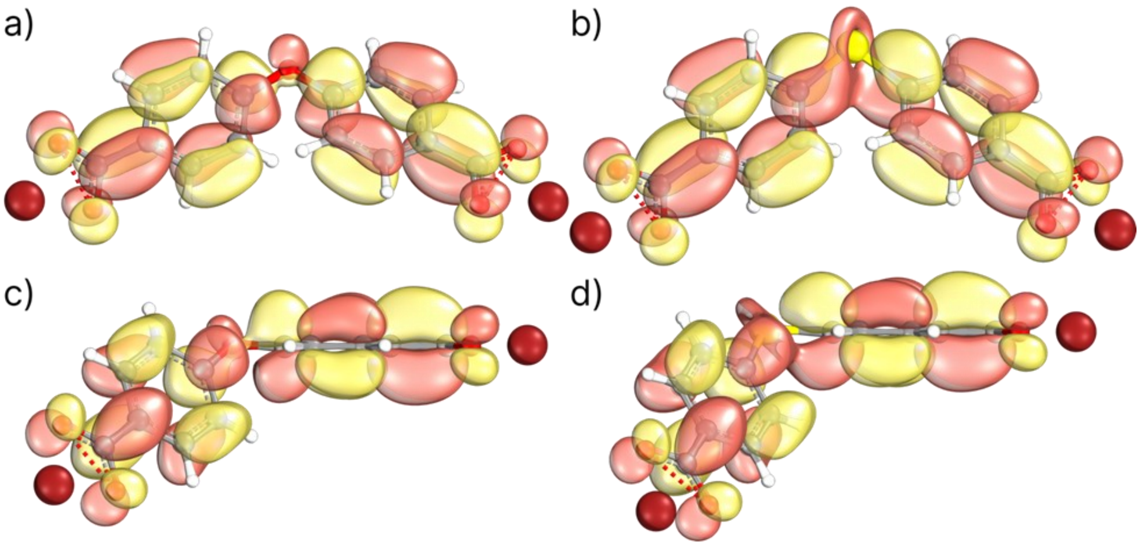

Figure S19 Visualization of LUMO orbitals for oba (a, c) and sba (b, d) linkers. Bottom images (c, d) represent alternative orientations for clarity.

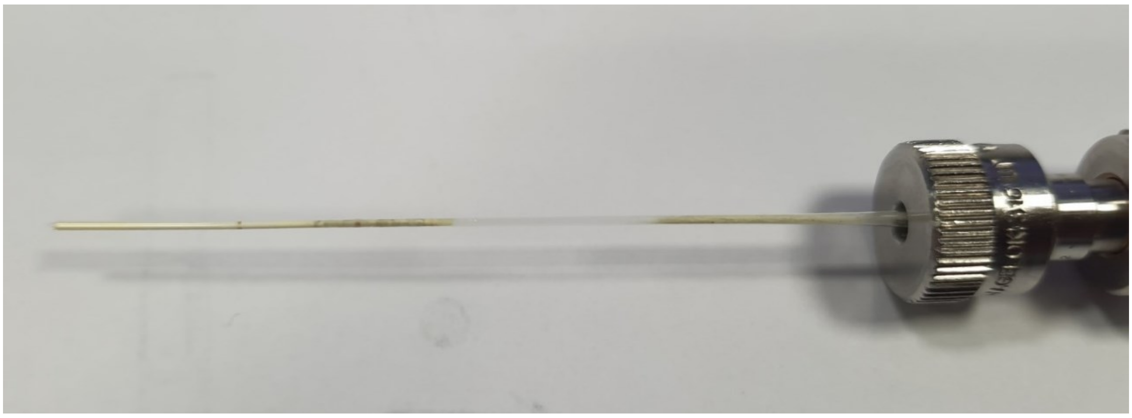

Figure S20 Capillary with loaded UAM-1O, after time-resolved PXRD measurement. Black dots indicate destruction of part of the sample.

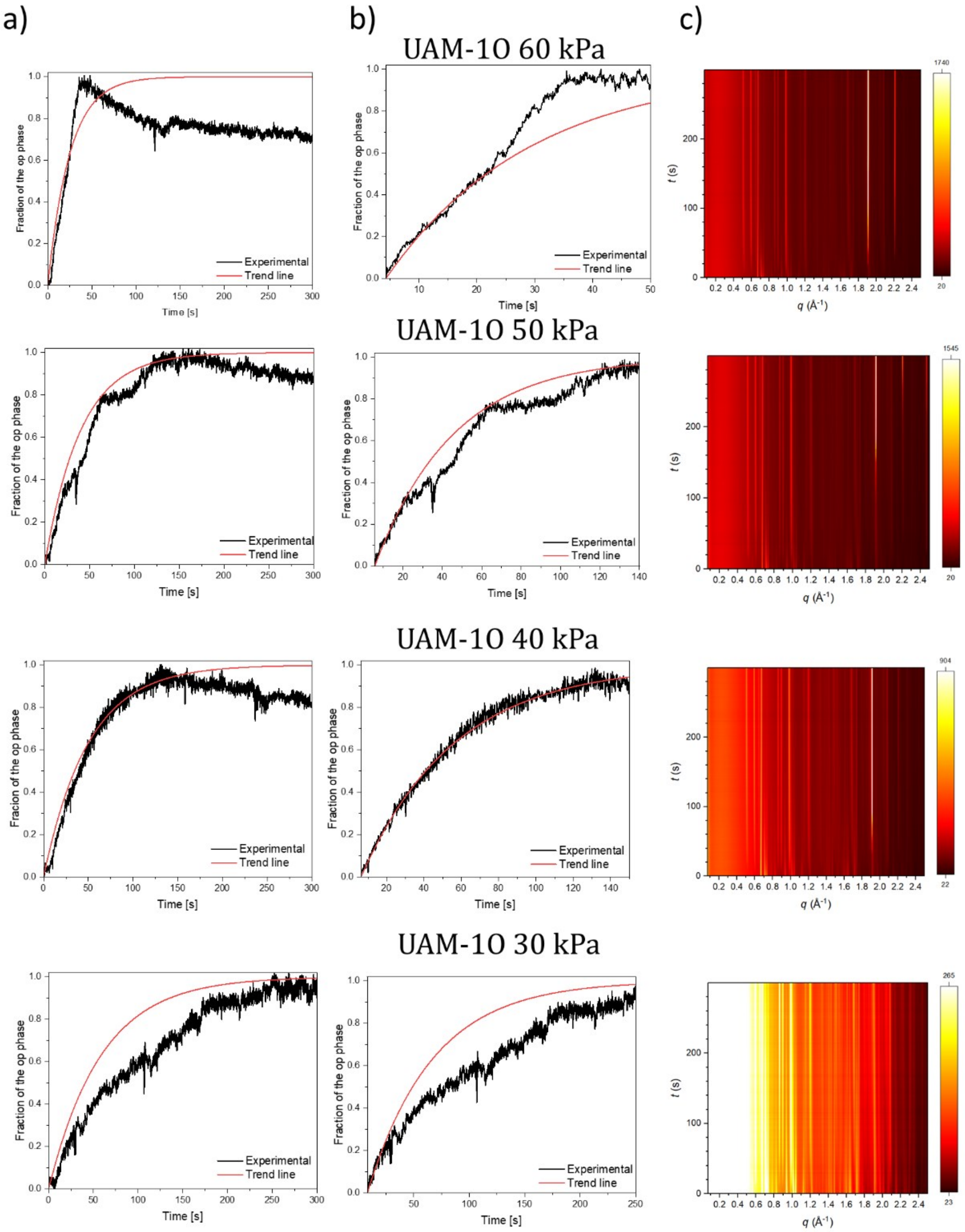

Figure S21 Fractions of the open pore phase for UAM-1O based on in situ synchrotron PXRD measurement normalized peaks intensity and the trend line was obtained by fitting the experimental data to the KJMA equation a) Full data; b) modified data by cutting ; c) Color maps of in situ PXRD patterns collected upon CO_2 adsorption on UAM-1O

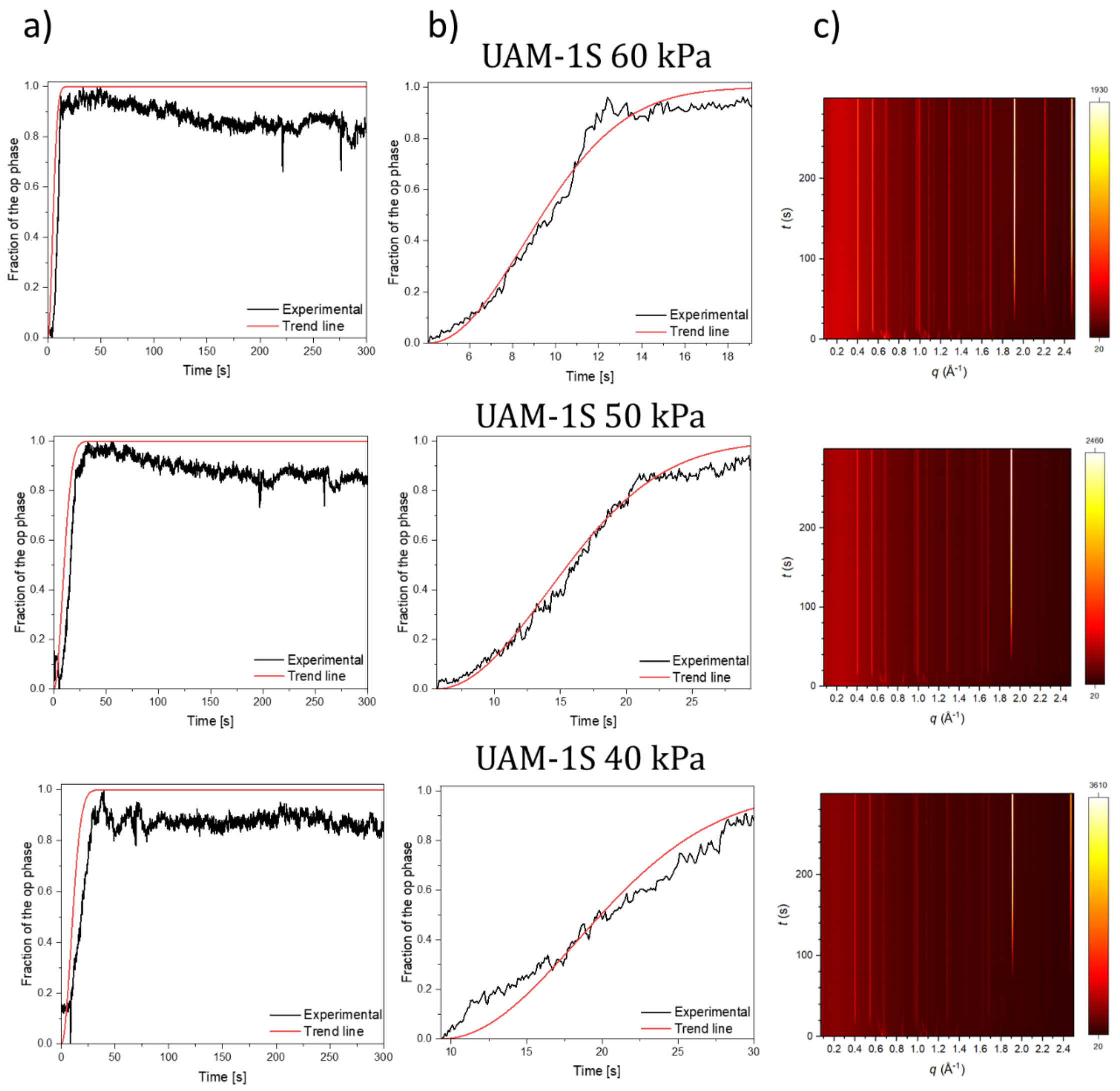

Figure S22 Fractions of the open pore phase for UAM-1S in different pressures and color maps of in situ PXRD patterns collected upon CO_2 adsorption (195 K).

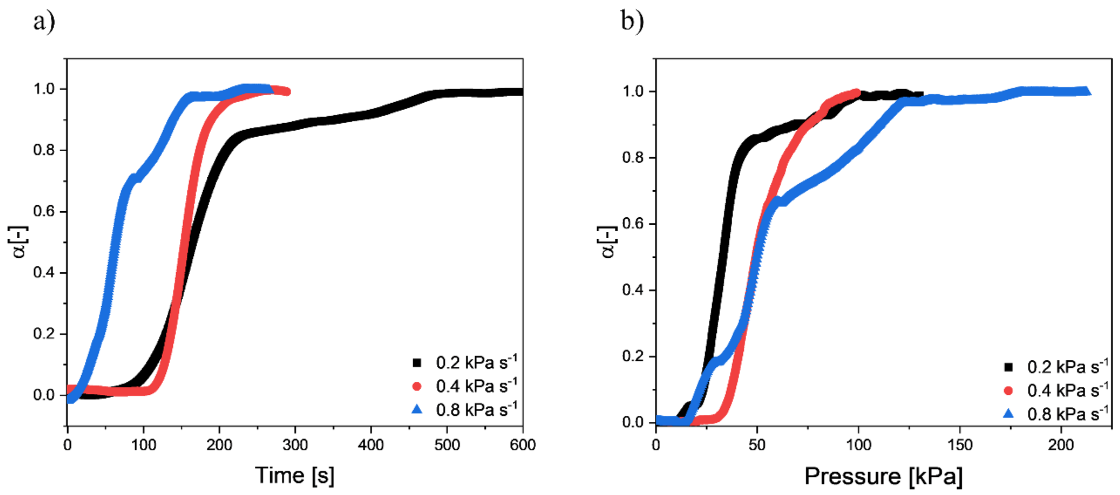

Figure S23 a) Time evolution of α at 195 K and 0.2, 0.4 and 0.8 kPa·s^{-1}. **b**) Pressure dependence of α in **a**.

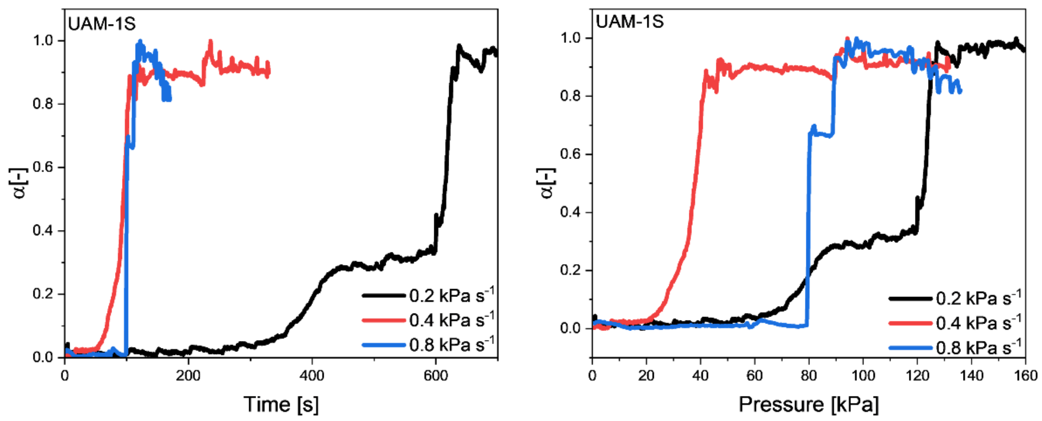

Figure S24. a) Time evolution of α at 195 K and 0.2, 0.4 and 0.8 kPa·s^{-1}. **b)** Pressure dependence of α in a.

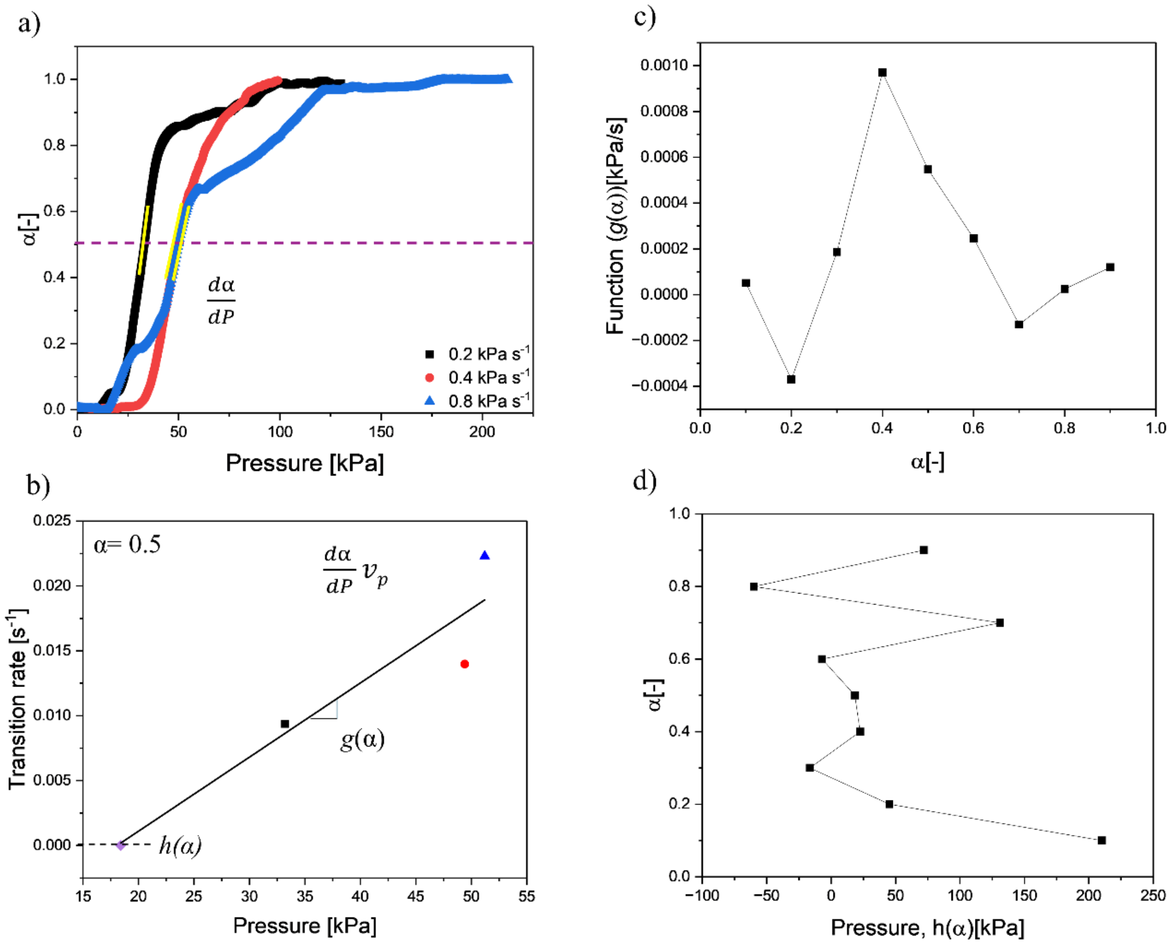

Figure S25 Attempt at deriving the structural transition rate model for UAM-1O according to Watanabe's work[12]. **a**) Pressure dependence of the structural transition rate at various α values. **a, b**) Example of analysis for α = 0.5, where the derivative values of α with respect to pressure are extracted from time-resolved in situ X-ray powder diffraction (TRXRD) results at 0.005, 0.2, 0.4, and 0.8 kPa s^{-1}. The dα/dP values are obtained by multiplying the pressurization rate. The solid line in panel b is a linear least-squares fit, where the slope represents the function g(α) and the x-intercept represents h(α). **c**) Relationship between the function g(α) obtained for different α values. **d**) Relationship between α and the function h(α) obtained for different α.

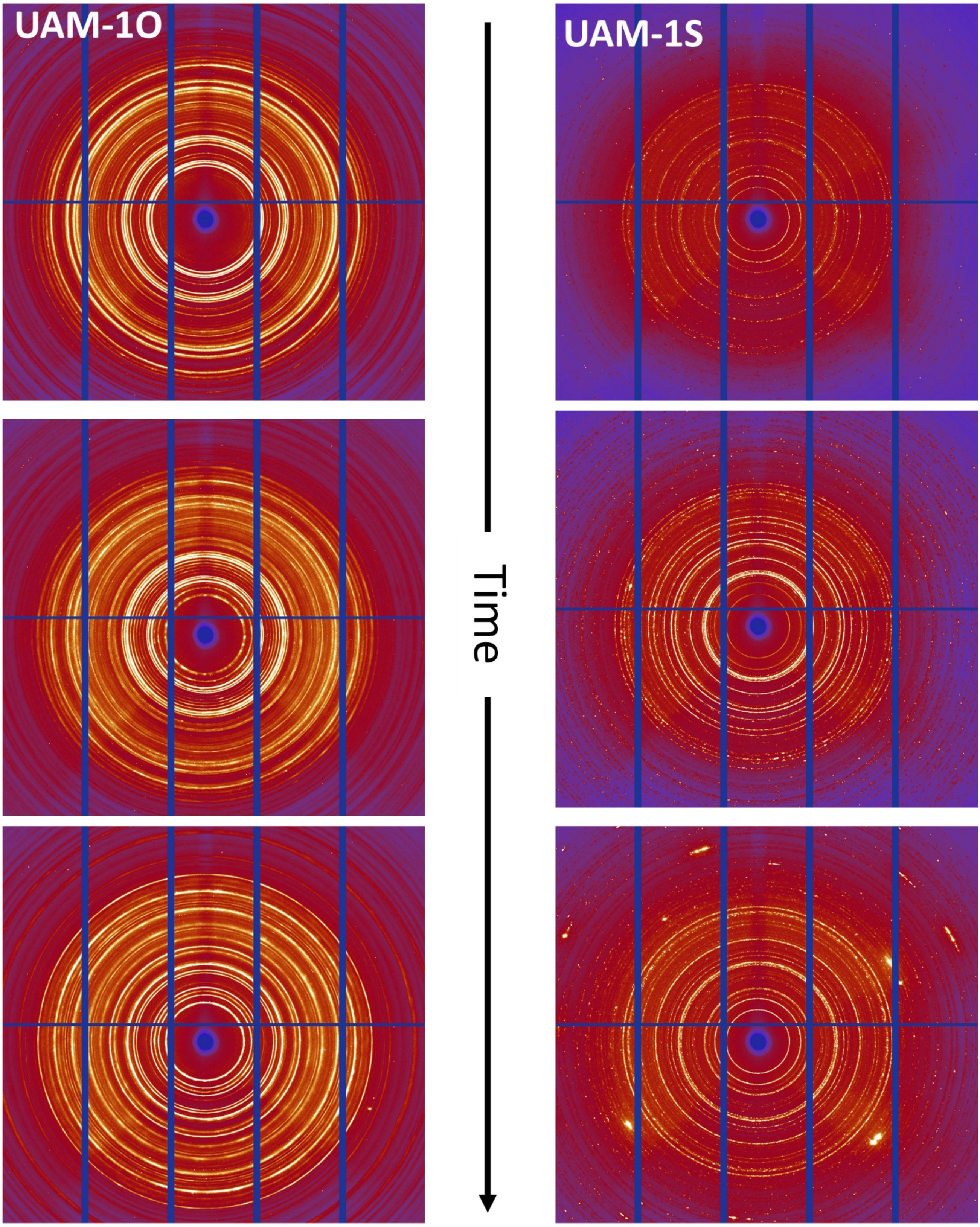

Figure S26 Diffraction images from time-resolved studies

Tables

Table 1 Summary of the microED data collection, reduction, and refinement statistics of the compounds.

	**UAM-1O cp**	**UAM-1S cp**
**CCDC**	2408974	2408973
**Chemical Formula**	$C_{21}H_{12}N_2O_5S_1Zn$	$C_{21}H_{12}N_2O_4S_2Zn$
**Tilt angle / tilt speed (º)**	0.5 / 1	
**Detector distance (mm)**	657	
**Temperature (K)**	81	
**Accelerating voltage (kV)**	200	
**Wavelength (Å)**	0.02508	
**Data reduction**		
**Space group**	*C2/c*	*C2/c*
**Unit cell *a, b, c* (Å)**	22.0, 10.2, 17.1	22.8, 10.1, 20.4
**Angles *α, β, γ* (º)**	90, 99.7, 90	90, 117.0, 90
**Volume ($Å^{-3}$)**	3782.4	4185.7
**Resolution (Å)**	0.70	0.70
**Total reflections**	14368	14634
**Unique reflections**	5661	4641
**Completeness (%)**	96.9	77.4
**Kinematical Refinement**		
**Reflections used (with $I > 2\sigma(I)$)**	5661 (2651)	4641 (4110)
**Parameters**	266	
***R1* [$I > 2\sigma(I)$]**	0.2470	0.1623
***wR2* [$I > 2\sigma(I)$]**	0.5256	0.3568
***R1* (all data)**	0.3379	0.4761
***wR2* (all data)**	0.5710	0.5869
GooF	1.489	0.9196
**Residual potential Max./min. ($Å^{-2}$)**	2.6/-1.3	1.5/-1.7

Table 2 The crystallographic data of metastable phase UAM-1O op.

UAM-1O(op)$_M$	
**CCDC**	2419835
**Empirical formula**	$C_{84}H_{48}N_8O_{20}S_4Zn_4$
**Formula weight**	1879.02
**Temperature/K**	135
**Crystal system**	Orthorhombic
**Space group**	*Pbcn*
**a/b/c [Å]**	31.2807 (4), 23.0755 (3), 15.8735 (2)
**α/ β/ γ [º]**	90
**Volume/Å^3**	11457.8 (3)
**Z**	4
**d [g/cm^3]**	1.089
**Radiation type**	Cu *K*α
**μ/mm^{-1}**	2.10
**F(000)**	3808.0
**Crystal size/mm**	0.39 × 0.11 × 0.08
**2Θ range for data collection/°**	4.758 to 152.728
**Index ranges**	$-34 \leq h \leq 39$, $-29 \leq k \leq 28$, $-19 \leq l \leq 19$
**Reflections collected**	60451
**Independent reflections**	11844 [R_{int} = 0.0373, R_{sigma} = 0.0278]
**Data/restraints/parameters**	11844/0/541
**Goodness-of-fit on F^2**	1.031
**Final R indexes [I>=2σ (I)]**	R_1 = 0.0619, wR_2 = 0.1800
**Final R indexes [all data]**	R_1 = 0.0843, wR_2 = 0.1972
**Largest diff. peak/hole /eÅ^{-3}**	1.33, −0.62

Table 3 Volume of equilibrium structures (V_{in}), their energy ($E_{V_{min}}$), EOS bulk modulus (B_0), and the derivative of bulk modulus with respect to pressure (B_0').

		V_{min} (Å^3)	$E_{V_{min}}$ (eV)	B_0 (GPa)	B_0'
**UAM-1O**	cp_1	8059.73	-4690.73	7.30	14.13
	op	11570.33	-4688.05	4.79	-18.03
	cp_2	8033.39	-4692.09		
**UAM-1S**	cp_1	9066.89	-4651.18	3.35	-11.62
	op	11353.30	-4649.27	4.02	14.94
	cp_2	8749.56	-4648.14		

Literature

(1) *(IUCr) XDS*. https://journals.iucr.org/d/issues/2010/02/00/dz5179/index.html (accessed 2024-10-31).

(2) *(IUCr) SHELXT – Integrated space-group and crystal-structure determination*. https://journals.iucr.org/a/issues/2015/01/00/sc5086/index.html (accessed 2024-10-31).

(3) Dolomanov, O. V.; Bourhis, L. J.; Gildea, R. J.; Howard, J. a. K.; Puschmann, H. OLEX2: A Complete Structure Solution, Refinement and Analysis Program. *J. Appl. Crystallogr.* **2009**, *42* (2), 339–341. https://doi.org/10.1107/S0021889808042726.

(4) Clark, R. C.; Reid, J. S. The Analytical Calculation of Absorption in Multifaceted Crystals. *Acta Crystallogr. A* **1995**, *51* (6), 887–897. https://doi.org/10.1107/S0108767395007367.

(5) CrysAlisPro Oxford Diffraction/Agilent Technologies UK Ltd., Yarnton, Oxfordshire, England. .

(6) Sheldrick, G. M. A Short History of SHELX. *Acta Crystallogr. A* **2008**, *64* (1), 112–122. https://doi.org/10.1107/S0108767307043930.

(7) Sheldrick, G. M. Crystal Structure Refinement with SHELXL. *Acta Crystallogr. Sect. C Struct. Chem.* **2015**, *71* (1), 3–8. https://doi.org/10.1107/S2053229614024218.

(8) Barbour, L. J. X-Seed — A Software Tool for Supramolecular Crystallography. *J. Supramol. Chem.* **2001**, *1* (4), 189–191. https://doi.org/10.1016/S1472-7862(02)00030-8.

(9) Spek, A. L. checkCIF Validation ALERTS: What They Mean and How to Respond. *Acta Crystallogr. Sect. E Crystallogr. Commun.* **2020**, *76* (1), 1–11. https://doi.org/10.1107/S2056989019016244.

(10) Dolomanov, O. V.; Bourhis, L. J.; Gildea, R. J.; Howard, J. a. K.; Puschmann, H. OLEX2: A Complete Structure Solution, Refinement and Analysis Program. *J. Appl. Crystallogr.* **2009**, *42* (2), 339–341. https://doi.org/10.1107/S0021889808042726.

(11) Kieffer, J.; Valls, V.; Blanc, N.; Hennig, C. New Tools for Calibrating Diffraction Setups. *J. Synchrotron Radiat.* **2020**, *27* (2), 558–566. https://doi.org/10.1107/S1600577520000776.

(12) Sakanaka, Y.; Hiraide, S.; Sugawara, I.; Uematsu, H.; Kawaguchi, S.; Miyahara, M. T.; Watanabe, S. Generalised Analytical Method Unravels Framework-Dependent Kinetics of Adsorption-Induced Structural Transition in Flexible Metal–Organic Frameworks. *Nat. Commun.* **2023**, *14* (1), 6862. https://doi.org/10.1038/s41467-023-42448-3.

(13) Rappe, A. K.; Casewit, C. J.; Colwell, K. S.; Goddard, W. A. I.; Skiff, W. M. UFF, a Full Periodic Table Force Field for Molecular Mechanics and Molecular Dynamics Simulations. *J. Am. Chem. Soc.* **1992**, *114* (25), 10024–10035. https://doi.org/10.1021/ja00051a040.

(14) Kresse, G.; Hafner, J. Ab Initio Molecular Dynamics for Liquid Metals. *Phys. Rev. B* **1993**, *47* (1), 558–561. https://doi.org/10.1103/PhysRevB.47.558.

(15) Kresse, G.; Furthmüller, J. Efficiency of Ab-Initio Total Energy Calculations for Metals and Semiconductors Using a Plane-Wave Basis Set. *Comput. Mater. Sci.* **1996**, *6* (1), 15–50. https://doi.org/10.1016/0927-0256(96)00008-0.

(16) Kresse, G.; Furthmüller, J. Efficient Iterative Schemes for Ab Initio Total-Energy Calculations Using a Plane-Wave Basis Set. *Phys. Rev. B* **1996**, *54* (16), 11169–11186. https://doi.org/10.1103/PhysRevB.54.11169.

(17) Perdew, J. P.; Burke, K.; Ernzerhof, M. Generalized Gradient Approximation Made Simple. *Phys. Rev. Lett.* **1996**, *77* (18), 3865–3868. https://doi.org/10.1103/PhysRevLett.77.3865.

(18) Grimme, S.; Antony, J.; Ehrlich, S.; Krieg, H. A Consistent and Accurate Ab Initio Parametrization of Density Functional Dispersion Correction (DFT-D) for the 94 Elements H-Pu. *J. Chem. Phys.* **2010**, *132* (15), 154104. https://doi.org/10.1063/1.3382344.

(19) Grimme, S.; Ehrlich, S.; Goerigk, L. Effect of the Damping Function in Dispersion Corrected Density Functional Theory. *J. Comput. Chem.* **2011**, *32* (7), 1456–1465. https://doi.org/10.1002/jcc.21759.

(20) Vanpoucke, D. E. P.; Lejaeghere, K.; Van Speybroeck, V.; Waroquier, M.; Ghysels, A. Mechanical Properties from Periodic Plane Wave Quantum Mechanical Codes: The Challenge of the Flexible Nanoporous MIL-47(V) Framework. *J. Phys. Chem. C* **2015**, *119* (41), 23752–23766. https://doi.org/10.1021/acs.jpcc.5b06809.

(21) Vinet, P.; Ferrante, J.; Rose, J. H.; Smith, J. R. Compressibility of Solids. *J. Geophys. Res. Solid Earth* **1987**, *92* (B9), 9319–9325. https://doi.org/10.1029/JB092iB09p09319.

(22) Sheppard, D.; Xiao, P.; Chemelewski, W.; Johnson, D. D.; Henkelman, G. A Generalized Solid-State Nudged Elastic Band Method. *J. Chem. Phys.* **2012**, *136* (7), 074103. https://doi.org/10.1063/1.3684549.

(23) *About TSASE: Transition State Library for ASE — TSASE: Transition-State Library for ASE*. https://theory.cm.utexas.edu/tsase/ (accessed 2025-01-21).

(24) Neese, F. Software Update: The ORCA Program System—Version 5.0. *WIREs Comput. Mol. Sci.* **2022**, *12* (5), e1606. https://doi.org/10.1002/wcms.1606.

(25) Grimme, S.; Hansen, A.; Ehlert, S.; Mewes, J.-M. r2SCAN-3c: A "Swiss Army Knife" Composite Electronic-Structure Method. *J. Chem. Phys.* **2021**, *154* (6), 064103. https://doi.org/10.1063/5.0040021.

(26) Knizia, G. Intrinsic Atomic Orbitals: An Unbiased Bridge between Quantum Theory and Chemical Concepts. *J. Chem. Theory Comput.* **2013**, *9* (11), 4834–4843. https://doi.org/10.1021/ct400687b.

(27) Knizia, G.; Klein, J. E. M. N. Electron Flow in Reaction Mechanisms—Revealed from First Principles. *Angew. Chem. Int. Ed.* **2015**, *54* (18), 5518–5522. https://doi.org/10.1002/anie.201410637.

(28) Roztocki, K.; Sobczak, S.; Smaruj, A.; Walczak, A.; Gołdyn, M.; Bon, V.; Kaskel, S.; Stefankiewicz, A. R. Tuning the Guest-Induced Spatiotemporal Response of Isostructural Dynamic Frameworks towards Efficient Gas Separation and Storage. *J. Mater. Chem. A* **2023**, *11* (35), 18646–18650. https://doi.org/10.1039/D3TA02167J.
